# Mobile Health Intervention in Patients With Type 2 Diabetes: A Randomized Clinical Trial

**DOI:** 10.1001/jamanetworkopen.2023.33629

**Published:** 2023-09-29

**Authors:** Ben S. Gerber, Alana Biggers, Jessica J. Tilton, Daphne E. Smith Marsh, Rachel Lane, Dan Mihailescu, JungAe Lee, Lisa K. Sharp

**Affiliations:** 1 Department of Population and Quantitative Health Sciences, University of Massachusetts Chan Medical School, Worcester; 2 Department of Medicine, College of Medicine, University of Illinois Chicago, Chicago; 3 Department of Pharmacy Practice, College of Pharmacy, University of Illinois Chicago, Chicago; 4 Center for Clinical and Translational Science, University of Illinois Chicago, Chicago; 5 Department of Endocrinology, Cook County Health, Chicago, Illinois; 6 Department of Biobehavioral Nursing Science, College of Nursing, University of Illinois Chicago, Chicago

## Abstract

**Importance:**

Clinical pharmacists and health coaches using mobile health (mHealth) tools, such as telehealth and text messaging, may improve blood glucose levels in African American and Latinx populations with type 2 diabetes.

**Objective:**

To determine whether clinical pharmacists and health coaches using mHealth tools can improve hemoglobin A_1c_ (HbA_1c_) levels.

**Design, Setting, and Participants:**

This randomized clinical trial included 221 African American or Latinx patients with type 2 diabetes and elevated HbA_1c_ (≥8%) from an academic medical center in Chicago. Adult patients aged 21 to 75 years were enrolled and randomized from March 23, 2017, through January 8, 2020. Patients randomized to the intervention group received mHealth diabetes support for 1 year followed by monitored usual diabetes care during a second year (follow-up duration, 24 months). Those randomized to the waiting list control group received usual diabetes care for 1 year followed by the mHealth diabetes intervention during a second year.

**Interventions:**

The mHealth diabetes intervention included remote support (eg, review of glucose levels and medication intensification) from clinical pharmacists via a video telehealth platform. Health coach activities (eg, addressing barriers to medication use and assisting pharmacists in medication reconciliation and telehealth) occurred in person at participant homes and via phone calls and text messaging. Usual diabetes care comprised routine health care from patients’ primary care physicians, including medication reconciliation and adjustment.

**Main Outcomes and Measures:**

Outcomes included HbA_1c_ (primary outcome), blood pressure, cholesterol, body mass index, health-related quality of life, diabetes distress, diabetes self-efficacy, depressive symptoms, social support, medication-taking behavior, and diabetes self-care measured every 6 months.

**Results:**

Among the 221 participants (mean [SD] age, 55.2 [9.5] years; 154 women [69.7%], 148 African American adults [67.0%], and 73 Latinx adults [33.0%]), the baseline mean (SD) HbA_1c_ level was 9.23% (1.53%). Over the initial 12 months, HbA_1c_ improved by a mean of −0.79 percentage points in the intervention group compared with −0.24 percentage points in the waiting list control group (treatment effect, −0.62; 95% CI, −1.04 to −0.19; *P* = .005). Over the subsequent 12 months, a significant change in HbA_1c_ was observed in the waiting list control group after they received the same intervention (mean change, −0.57 percentage points; *P* = .002), while the intervention group maintained benefit (mean change, 0.17 percentage points; *P* = .35). No between-group differences were found in adjusted models for secondary outcomes.

**Conclusions and Relevance:**

In this randomized clinical trial, HbA_1c_ levels improved among African American and Latinx adults with type 2 diabetes. These findings suggest that a clinical pharmacist and health coach–delivered mobile health intervention can improve blood glucose levels in African American and Latinx populations and may help reduce racial and ethnic disparities.

**Trial Registration:**

ClinicalTrials.gov Identifier: NCT02990299

## Key Points

**Question:**

Does a clinical pharmacist and health coach–delivered mobile health intervention improve blood glucose levels in African American and Latinx adults with type 2 diabetes and hemoglobin A_1c_ levels of 8% or higher?

**Findings:**

In this randomized clinical trial of 221 adults with type 2 diabetes, hemoglobin A_1c_ decreased by a mean of 0.79 percentage points in the intervention group over 1 year. This decrease was significantly different from the mean of 0.24 percentage points observed in the waiting list control group.

**Meaning:**

These findings suggest that a clinical pharmacist and health coach–delivered mobile health intervention can improve blood glucose levels in African American and Latinx populations and may help reduce racial and ethnic disparities.

## Teaser

This randomized clinical trial assesses whether a clinical pharmacist and health coach–delivered mobile health intervention can improve hemoglobin A_1c_ levels in adult African American and Latinx patients with type 2 diabetes.

## Introduction

Pharmacists are increasingly contributing to diabetes management and cardiovascular disease risk reduction.^1^ Office-embedded clinical pharmacists can provide comprehensive medication management,^2^ addressing adverse effects and drug interactions,^3^ assisting in medication taking,^4^ and intensifying therapy.^5^ While intensification of therapy through prescriptive authority varies in the US,^6^ such collaborative action with physicians can improve outcomes.^7^ However, there remains limited integration of clinical pharmacists in community settings due to lack of reimbursement, clinician acceptance, time, and resources.^8^ These limitations represent substantial barriers to pharmacists’ ability to reach and effectively care for racial and ethnic minority populations.

Previous work^9,10^ has investigated clinical pharmacists partnering with community health workers (health coaches) to engage diverse populations with type 2 diabetes (T2D). Studies have focused on African American and Latinx populations with T2D who experienced higher mean hemoglobin A_1c_ (HbA_1c_) levels than non-Latinx White populations.^11,12,13,14^ There is evidence that health coaches alone can modestly improve HbA_1c_,^15^ increase short-term diabetes understanding, lower diabetes distress, and improve self-care.^16,17,18^ Health coaches can potentially extend pharmacist services, support medication use or changes in therapy, and encourage healthy eating and physical activity behaviors with awareness of sociocultural issues.^19,20^

Mobile health (mHealth), including telehealth and text messaging, may further enhance both pharmacist and health coach activities. Pharmacists have used videoconferencing in chronic disease management to improve health care access.^21,22,23,24^ While telehealth can improve diabetes management, few studies have had meaningful representation of African American and Latinx patients and, to our knowledge, no studies have investigated the facilitation of telehealth access in this context.^25^ Health coaches visiting patients at home may participate in 3-way videoconference calls, allowing them to facilitate and collaborate with both the pharmacist and patient simultaneously. Between these encounters, health coaches can engage patients through text messaging to reinforce self-management.^26,27^ This approach may be more effective than text messaging support alone, which has not shown improvement in HbA_1c_ beyond a short duration.^26^

This study evaluated a clinical pharmacist and health coach–delivered mHealth-based model of care through a randomized clinical trial. The intervention targeted urban African American and Latinx populations with T2D and HbA_1c_ levels of 8.0% or higher. We hypothesized that individuals receiving the intervention would have a greater reduction in HbA_1c_ than those receiving usual diabetes care. We specifically designed the study to allow for a waiting list control group to receive the intervention after a 12-month delay so we could evaluate consequent changes in outcomes. Additionally, those randomized to receive the intervention first were followed up for an extra 12 months to assess the maintenance of any improvement made.

## Methods

### Study Design

**Figure 1.**
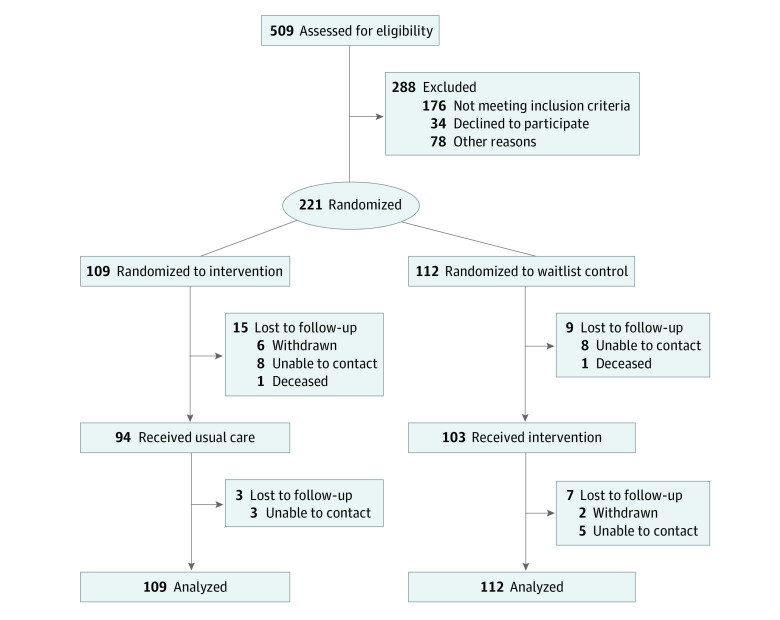
Study Enrollment Flowchart

This 2-arm parallel randomized clinical trial was performed in academic primary care clinics in Chicago and included patients with T2D. The trial protocol is available in Supplement 1 and has been revised for publication.^28^ Patients were randomized 1:1 to receive either the mHealth diabetes support intervention for 1 year followed by monitored usual diabetes care during a second year (intervention group) or usual diabetes care for 1 year followed by the mHealth diabetes support intervention during a second year (waiting list control group) (Figure 1). The study was approved by the University of Illinois Chicago Institutional Review Board. All participants provided written informed consent. This study followed the Consolidated Standards of Reporting Trials (CONSORT) reporting guideline for randomized clinical trials.^29^

### Setting and Participants

Patients were enrolled through primary care clinics within the University of Illinois Hospital and Health Sciences System (UI Health) from March 23, 2017, through January 8, 2020. Patients were followed up for 24 months (until January 8, 2022). UI Health contains academic and federally qualified health centers (FQHCs) that provide community-oriented care to Chicago neighborhoods. Eligibility was initially assessed in person at the time of an office visit or by telephone. Inclusion criteria were (1) self-identifying as African American or Black race or Hispanic or Latinx ethnicity, (2) being between ages 21 and 75 years, (3) receiving primary care at the clinical site for at least 1 year, (4) having verbal fluency in the English or Spanish language, (5) having a mobile phone and text messaging plan, and (6) agreeing to receive home visits by a health coach. The presence of T2D and at least 1 recorded HbA_1c_ level of 8.0% or higher in the past 6 months (not necessarily the most recent recorded level) were confirmed by electronic medical record (EMR) review. Exclusion criteria were (1) being unable to verbalize comprehension of the study or impaired decision-making (eg, dementia), (2) living outside of Chicago (≥3 months per year), (3) having a household member who was already participating in the study, (4) planning to move from the Chicago area within the next year, (5) being pregnant or trying to get pregnant, (6) being unable to send or read text messages on mobile phone, and (7) having a history of receiving or planning to receive gastric bypass or transplant surgery. Of 509 patients assessed for eligibility, 221 provided consent and were randomized (eTable 1 in Supplement 2).

### Randomization

We used a computerized random number generator integrated into the Research Electronic Data Capture (REDCap) platform and stratified by sex (male or female), race and ethnicity (African American or Latinx), and clinical location (10 sites). After participants completed baseline data collection assessments, a coordinator informed them of their randomization assignments. Participants who withdrew from the study before completing baseline assessments and were not randomized were excluded from data analyses. After randomization, all participants received a list of clinic resources with names and telephone numbers (eg, social worker or clinical pharmacist) along with a low-literacy diabetes education guidebook (*Living with Diabetes: An Everyday Guide for You and Your Family* published by the American College of Physicians^30^ in English or Spanish).

### Intervention

A detailed description of the intervention implementation can be found in the published version of the trial protocol.^28^ Pharmacists followed a standardized protocol that included medication reconciliation and ongoing assessment of medication changes, identification of therapeutic goals for HbA_1c_ and blood pressure with patients’ primary care physicians (PCPs), review of home glucose and/or blood pressure logs, formulation of a protocol-based care plan, and EMR documentation. In addition, pharmacists provided education related to medication taking, drug interactions, and adverse effects; promoted basic lifestyle modifications; and supported medication-taking behavior through aids such as pill boxes and low-literacy medication lists. Pharmacists actively intensified therapy using the UI Health guideline-based pharmacist diabetes management protocol and consulted with PCPs in advance if necessary.

Pharmacist encounters occurred remotely via telehealth (eFigure in Supplement 2). Before the COVID-19 pandemic (defined as before March 16, 2000), health coaches inside the patient’s home facilitated the videoconference via an internet-enabled computer tablet (iPad Air 2 or fifth generation; Apple Inc) with a cellular plan. During the COVID-19 pandemic (defined as March 16, 2000, to end of study period on January 8, 2022), 3-way calling (audio or video) was conducted. Pharmacist encounters ranged from 30 to 60 minutes based on the treatment plan and occurred at least every 2 to 3 months. Pharmacists reviewed patient medical records, including laboratory results, progress notes, and medication lists; documented telehealth encounters; and communicated with PCPs through EMR secure messaging and progress note forwarding. Health coaches scheduled appointments based on the pharmacist’s clinic schedule. Telehealth was conducted using the VSee platform (VSee), which adhered to the Health Insurance Portability and Accountability Act of 1996.^31^

African American and Latinx health coaches were hired and completed extensive study-specific training. Health coaches introduced themselves to participants receiving the intervention, contacted them at least monthly, and conducted home visits (targeting every other month) and phone calls, including facilitation of telehealth with pharmacists on alternating visits. The health coaches addressed medication use (eg, reviewing pill bottle labels), assisted pharmacists in medication reconciliation, compiled home glucose monitoring data, and reinforced pharmacists’ recommendations. Health coaches alerted pharmacists to extremely high or low home glucose levels, patient questions, and discrepancies discovered in management. In addition, health coaches provided diabetes self-management education, support with information sharing, psychosocial and goal-based behavioral support, and coordination of care. During home visits, health coaches monitored glucose and blood pressure levels, which were shared with pharmacists. When appropriate, health coaches also used the *Living Well with Diabetes* (*Viviendo Bien con Diabetes*)^32^ electronic book (for iPad) to provide interactive multimedia education. Similar to pharmacists, coaches documented patient encounters in the EMR and summarized their activities in a REDCap tracking form.

Health coaches used text messaging on study telephones to engage patients between visits. Coaches used a custom software application (mytapp; developed by B.G.) to schedule future text messages, including appointment and medication reminders and messages to motivate patients toward self-management goals.^33^ The health coaches monitored patient replies to messages in real time. Fidelity to all intervention components was assessed weekly by study investigators (B.G., A.B, and L.S.) monitoring health coach activities, including text messaging, home visits, phone calls, and telehealth.

### Usual Care

For usual diabetes care, participants received routine health care from their PCPs. This care included medication reconciliation and adjustment.

### Measurement

Study measurements were collected by trained researchers at baseline, 6 months, 12 months, 18 months, and 24 months. Because of the nature of the intervention, it was not possible to blind patients, interventionists (pharmacists or health coaches), or researchers to intervention assignments. However, HbA_1c_ measurement (primary outcome) was completed independently by laboratories without knowledge of intervention assignments.

Hemoglobin A_1c_ values and fasting lipid profiles (including total cholesterol, high- and low-density lipoprotein cholesterol, and triglyceride levels) were obtained via phlebotomy at the medical center (Alverno Laboratories; Hammond, Indiana) before the COVID-19 pandemic and via home fingerstick testing (Home Access; Hoffman Estates, Illinois) during the early COVID-19 pandemic. Standardized protocols were used to measure height, weight, and blood pressure. Questionnaires were administered via interview, with responses entered directly into the REDCap system. These questionnaires included health-related quality of life (EuroQol 5-dimension survey^34^), diabetes behaviors (revised Summary of Diabetes Self-Care Activities measure^35^), diabetes distress (brief Diabetes Distress Scale^36^), depression (9-item Patient Health Questionnaire^37^), social support (assessment of the amount of total support received and satisfaction with support from family, friends, and the health care team^38^), self-efficacy (Stanford Self-Efficacy for Diabetes scale^39^), health literacy (brief 3-item screening^40,41^), and medication-taking behavior (self-rating question: “Over the past month, what percent of the time did you take all your diabetes medication as prescribed?”^42,43^). Electronic medical record queries provided prescription and clinic visit data for participants. Medication intensification for blood glucose management was defined as an increase in dose or number of therapeutic classes (with prescriptions written within 12 months before enrollment serving as baseline).

### Statistical Analysis

The sample size calculation was powered to detect the primary outcome of HbA_1c_ level. A systematic review of published studies^44^ suggested that successful educational programs lowered HbA_1c_ levels by 0.4 to 1.7 percentage points. We estimated a mean baseline HbA_1c_ level of 10.0% with an SD of 1.8% and an effect size of 0.56. The cross-time correlation was estimated to be 0.30 with a compound symmetry structure. We adjusted for clustering and assumed an intraclass correlation coefficient of 0.01 with 5 clusters. This adjustment yielded a design effect of 1.34. A 2-sided α = .05 and 80% power were assumed. Allowing for a 20% study withdrawal rate, 220 patients were required.

We followed international guidelines for analysis and reporting of clinical trial intention-to-treat principles. To test group differences in HbA_1c_ levels and other continuous outcomes, we used linear mixed-effects models for repeated measures over time within a 2-group 2-period framework. Each model included fixed terms for design effects (treatment, year, and measurement within year), random participant intercepts, and compound symmetry covariance. We did not find evidence of carryover effects (no carryover terms were included in the final model). Other explanatory variables included baseline blocking variables (site, sex, and race and ethnicity), age, and insurance status. Additional covariates and an indicator variable to reflect whether a measure was taken before or during the COVID-19 pandemic were considered; however, because the additional covariates and indicator variable did not improve the final model, they were excluded. We used 2-tailed *P* < .05 as the threshold to identify statistically significant differences. Between-group differences in outcomes and within-group change over time were evaluated by a priori contrasts with Holm-Bonferroni adjustment for multiple testing.^45^ We also tested whether baseline characteristics, diabetes behaviors, and health coach engagement (ie, number of encounters) were associated with HbA_1c_ change. Differences in the primary outcome were assessed for each group independently during the second year using a 1-sample *t* test (complete cases). In exploratory analyses, we calculated Spearman correlations between diabetes behaviors and change in HbA_1c_ level.

The primary analysis included all available data for each outcome. Likelihood-based mixed-effects models produce valid inferences in the presence of data missingness provided that the missing data mechanism is ignorable, and these models are appropriate for analyzing incomplete repeated-measures data in randomized clinical trials. Given the presence of missing data, we conducted 2 sensitivity analyses. First, we repeated the primary analysis using a completer data set composed of participants with HbA_1c_ values at baseline, month 12, and month 24. We then performed multiple imputation of missing values in 20 data sets by fully conditional specification conclusions and repeated the analysis. Additionally, we provided a multiplicity adjustment *P* value,^45^ treating all outcome variables simultaneously. Results of these sensitivity analyses were similar, and conclusions did not change; only the primary results were reported in this article. Data were analyzed using R software, version 4.3.0 (R Foundation for Statistical Computing).

## Results

### Participants and Retention

Among 221 participants, the mean (SD) age at baseline was 55.2 (9.5) years; 154 participants (69.7%) were women, and 67 (30.3%) were men. A total of 148 participants (67.0%) self-reported being of Black or African American race, and 73 (33.0%) of Hispanic or Latinx ethnicity (Table 1). The mean (SD) HbA_1c_ value at baseline was 9.23% (1.53%). At 12 months, 84 of 109 participants (77.1%) in the intervention group (intervention first, then usual care) and 99 of 112 (88.4%) in the waiting list control group (usual care first, then intervention) completed HbA_1c_ measurement. At 24 months, 80 of 109 participants (73.4%) in the intervention group and 90 of 112 (80.4%) in the waiting list control group completed HbA_1c_ measurement. There was comparable contact with pharmacists and health coaches in both groups. However, health coaches conducted more home and clinic visits in the intervention group and more phone calls in the waiting list control group (secondary to COVID-19 restrictions) (eTable 2 in Supplement 2). More health coach contact occurred among African American patients (mean [SD], 7.2 [4.0] encounters) vs Latinx patients (mean [SD], 5.0 [3.2] encounters; *P* < .001).

**Table 1.** Baseline Characteristics, Overall and Stratified by Randomization Group

Characteristic	Participants, No. (%)		
	Overall (N = 221)	Intervention group (n = 109)^a^	Waiting list control group (n = 112)^b^
Age, mean (SD), y	55.2 (9.5)	56.0 (9.3)	54.5 (9.6)
Diabetes duration, mean (SD), y	12.7 (7.8)	13.1 (7.7)	12.3 (7.9)
Race and ethnicity^c^			
Black or African American	148 (67.0)	72 (66.1)	76 (67.9)
Latinx or Hispanic	73 (33.0)	37 (33.9)	36 (32.1)
Sex			
Female	154 (69.7)	77 (70.6)	77 (68.8)
Male	67 (30.3)	32 (29.4)	35 (31.3)
Language preference			
English	184 (83.3)	87 (79.8)	97 (86.6)
Spanish	37 (16.7)	22 (20.2)	15 (13.4)
Annual income, $			
<10 000	74 (33.5)	36 (33.0)	38 (33.9)
10 000-19 999	45 (20.4)	26 (23.9)	19 (17.0)
20 000-29 999	28 (12.7)	16 (14.7)	12 (10.7)
30 000-39 999	17 (7.7)	10 (9.2)	7 (6.3)
40 000-49 999	12 (5.4)	0	12 (10.7)
50 000-59 999	14 (6.3)	8 (7.3)	6 (5.4)
60 000-69 999	5 (2.3)	3 (2.8)	2 (1.8)
≥70 000	22 (10.0)	8 (7.3)	14 (12.5)
Declined to answer	4 (1.8)	2 (1.8)	2 (1.8)
Educational level			
Less than high school	55 (24.9)	29 (26.6)	26 (23.2)
High school diploma or GED certificate	55 (24.9)	33 (30.3)	22 (19.6)
Some college, 2-y certificate, or associate’s degree	67 (30.3)	26 (23.9)	41 (36.6)
College degree	24 (10.9)	13 (11.9)	11 (9.8)
Some graduate school	6 (2.7)	3 (2.8)	3 (2.7)
Graduate degree	13 (5.9)	5 (4.6)	8 (7.1)
Other	1 (0.5)	0	1 (0.9)
Health status			
Poor	25 (11.3)	8 (7.3)	17 (15.2)
Fair	105 (47.5)	58 (53.2)	47 (42.0)
Good	80 (36.2)	38 (34.9)	42 (37.5)
Very good	9 (4.1)	5 (4.6)	4 (3.6)
Excellent	2 (0.9)	0	2 (1.8)
Insurance			
None	13 (5.9)	8 (7.3)	5 (4.5)
Public	139 (62.9)	70 (64.2)	69 (61.6)
Private	66 (29.9)	30 (27.5)	36 (32.1)
Other	3 (1.4)	1 (0.9)	2 (1.8)
Health literacy, mean (SD)^d^	5.6 (3.1)	5.8 (3.1)	5.4 (3.0)

Abbreviation: GED, general educational development.

^a^ The intervention group received the clinical pharmacist and health coach–delivered mobile health intervention in year 1, then usual diabetes care in year 2.

^b^ The waiting list control group received usual diabetes care in year 1, then the clinical pharmacist and health coach–delivered mobile health intervention in year 2.

^c^ Self-identified race and ethnicity as Black or African American or Hispanic or Latinx were study inclusion criteria.

^d^ Based on a brief 3-item screening (range, 3-15, with lower scores indicating better health literacy).^40,41^

### Hemoglobin A_1c_ Outcomes

**Figure 2.**
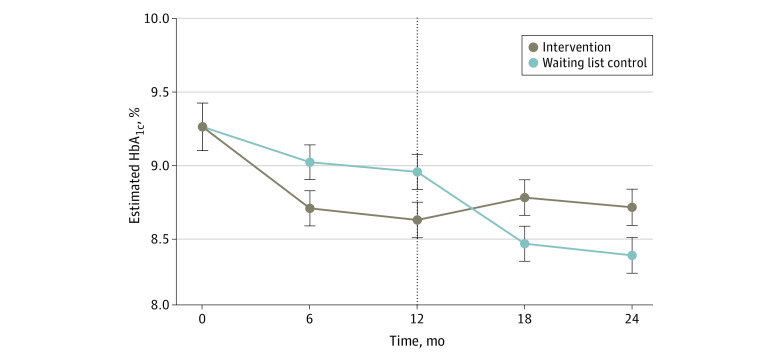
Comparison of Estimated Hemoglobin A_1c_ (HbA_1c_) Levels for Intervention and Waiting List Control Groups Over Time

In the intention-to-treat analysis, we found a significant improvement in mean HbA_1c_ of −0.79 percentage points in the intervention group compared with −0.24 percentage points in the waiting list control group over 12 months (treatment effect, −0.62; 95% CI, −1.04 to −0.19; *P* = .005). Furthermore, we observed a significant change in HbA_1c_ for the waiting list control group receiving the intervention during the subsequent 12 months (mean change, −0.57 percentage points; *P* = .002), while the intervention group maintained benefit (mean change, 0.17 percentage points; *P* = .35). Mean HbA_1c_ values over time for the 2 treatment groups are shown in Figure 2. After controlling for baseline covariates, the intervention treatment remained significant between groups. Similar results were found in the per protocol analysis and the analysis using multiple imputed data sets. No dose-response relationships were identified based on pharmacist and health coach encounters, and no heterogeneity of treatment effect was found based on race and ethnicity.

### Secondary Outcomes

Findings from linear mixed-effects models for secondary outcomes are reported in Table 2. Descriptive statistics for the secondary outcomes are available in eTable 3 in Supplement 2. We found unadjusted treatment effects for change in both diabetes self-efficacy (overall treatment effect, 0.22; *P* = .01) and social support (overall treatment effect, 0.46; *P* = .01). The mean high-density lipoprotein cholesterol level increased in the intervention group who received the intervention in year 1 but decreased in the waiting list control group who received the intervention in year 2 (overall treatment effect, −0.88; *P* = .03). However, there were no significant effects in any secondary outcome–adjusted models, including low-density lipoprotein cholesterol, systolic and diastolic blood pressure, body mass index (calculated as weight in kilograms divided by height in meters squared), medication-taking behavior, diabetes-related behaviors, diabetes distress, depression, and quality of life. Furthermore, no effects were observed in medication intensification or number of clinic visits (eTable 4 in Supplement 2). However, in exploratory analyses, we found associations between diabetes behaviors (medication taking, general diet, exercise, and blood glucose testing) and change in HbA_1c_ with the intervention (eTable 5 in Supplement 2). There were no adverse events related to study procedures. We estimated the cost for a health coach and clinical pharmacist to be approximately $2370 annually per patient (assuming a caseload of 30 patients).

**Table 2.** Results of Linear Mixed-Effects Model of Outcomes at 12 and 24 Months

Outcome and time, mo	Linear mixed-effects model by year and all time points^a^						
	LS mean (SE)		Treatment effect				
	Intervention group^b^	Waiting list control group^c^	By year	*P* value	Overall	*P* value	Adjusted *P* value^d^
Hemoglobin A_1c_, %							
12	8.33 (0.30)	8.95 (0.29)	−0.62	.005	NA	NA	NA
24	8.76 (0.38)	8.68 (0.38)	−0.08	.76	−0.32	<.001	.002
Systolic blood pressure, mm Hg							
12	131.16 (3.19)	135.03 (3.20)	−3.88	.07	NA	NA	NA
24	129.46 (3.73)	129.43 (3.76)	−0.04	.99	−2.07	.08	.93
Diastolic blood pressure, mm Hg							
12	79.04 (1.78)	80.63 (1.79)	−1.59	.18	NA	NA	NA
24	78.42 (1.90)	77.93 (1.92)	−0.49	.71	−0.78	.16	>.99
Total cholesterol, mg/dL							
12	158.35 (8.41)	165.62 (8.29)	−7.27	.24	NA	NA	NA
24	156.19 (10.71)	155.17 (10.62)	−1.02	.88	−2.09	.32	>.99
HDL cholesterol, mg/dL							
12	45.01 (2.10)	44.79 (2.07)	0.22	.88	NA	NA	NA
24	47.16 (2.78)	43.77 (2.76)	−3.39	.06	−0.88	.03	.38
LDL cholesterol, mg/dL							
12	85.33 (7.16)	89.71 (7.06)	−4.38	.40	NA	NA	NA
24	80.79 (8.45)	80.65 (8.38)	−0.14	.98	−1.07	.53	>.99
Triglycerides, mg/dL							
12	147.69 (22.96)	173.48 (22.63)	−25.79	.13	NA	NA	NA
24	139.27 (26.15)	156.55 (25.94)	17.28	.31	−3.12	.58	>.99
BMI^e^							
12	33.44 (1.95)	35.02 (1.96)	−1.57	.24	NA	NA	NA
24	33.24 (2.12)	36.54 (2.13)	3.31	.02	0.10	.34	>.99
Diabetes distress^f^^,^^g^							
12	2.07 (0.24)	2.37 (0.23)	−0.29	.09	NA	NA	NA
24	1.99 (0.28)	2.17 (0.28)	0.18	.32	−0.04	.54	>.99
Diabetes self-efficacy^h^^,^^i^							
12	7.27 (0.32)	7.06 (0.31)	0.22	.34	NA	NA	NA
24	7.43 (0.39)	7.53 (0.39)	0.10	.68	0.22	.01	.18
Depression^j^^,^^k^							
12	2.85 (0.92)	3.62 (0.91)	−0.77	.25	NA	NA	NA
24	2.53 (1.03)	2.94 (1.03)	0.41	.54	−0.08	.69	>.99
Diabetes social support^l^							
12	16.35 (0.76)	15.5 (0.74)	0.85	.12	NA	NA	NA
24	17.54 (0.77)	17.9 (0.76)	0.37	.46	0.46	.01	.18
Medication taking^m^							
12	89.85 (3.22)	85.39 (3.16)	4.47	.05	NA	NA	NA
24	86.56 (3.78)	82.79 (3.76)	−3.78	.13	0.75	.50	>.99
Diabetes self-care, diet score^n^							
12	4.37 (0.36)	4.32 (0.36)	0.05	.84	NA	NA	NA
24	4.09 (0.37)	4.39 (0.37)	0.30	.23	0.18	.10	>.99
Diabetes self-care, exercise score^n^							
12	2.57 (0.37)	2.32 (0.36)	0.25	.34	NA	NA	NA
24	2.56 (0.43)	2.31 (0.42)	−0.26	.36	0.06	.62	>.99
Diabetes self-care, glucose testing score^n^^,^^o^							
12	4.56 (0.46)	3.92 (0.45)	0.64	.06	NA	NA	NA
24	4.28 (0.54)	4.37 (0.54)	0.09	.79	0.31	.02	.33
Quality of life^p^							
12	77.52 (3.58)	75.38 (3.52)	2.14	.40	NA	NA	NA
24	71.98 (4.23)	70.09 (4.20)	−1.89	.49	0.52	.66	>.99

Abbreviations: BMI, body mass index (calculated as weight in kilograms divided by height in meters squared); HDL, high-density lipoprotein; LDL, low-density lipoprotein; LS, least squares; NA, not applicable.

SI conversion factors: To convert percentage of total HbA_1c_ to proportion of total HbA_1c_, multiply by 0.01; total cholesterol, HDL cholesterol, and LDL cholesterol to millimoles per liter, multiply by 0.0259; triglycerides to millimoles per liter, multiply by 0.0113.

^a^ The model included treatment, year, time (visit), site, sex, race and ethnicity, insurance status, and age. Random participant intercepts and compound symmetric covariance matrix were used.

^b^ The intervention group received the clinical pharmacist and health coach–delivered mobile health intervention in year 1, then usual diabetes care in year 2.

^c^ The waiting list control group received usual diabetes care in year 1, then the clinical pharmacist and health coach–delivered mobile health intervention in year 2.

^d^ Multiple-testing *P* values were adjusted using the Holm-Bonferroni method.^45^

^e^ Values are missing for 3 participants in the intervention group and 5 in the waiting list control group.

^f^ Assessed by the brief Diabetes Distress Scale (range, 1-6, with mean item score ≥3 indicating moderate distress).^36^

^g^ Values are missing for 1 participant in the waiting list control group.

^h^ Assessed by the Stanford Self-Efficacy for Diabetes scale (range, 1-10, with higher scores indicating greater self-efficacy).^39^

^i^ Values are missing for 1 participant in the waiting list control group.

^j^ Assessed by the 9-item Patient Health Questionnaire (range, 0-27, with scores of 5 indicating mild depression, 10 indicating moderate depression, 15 indicating moderately severe depression, and 20 indicating severe depression).^37^

^k^ Values are missing for 1 participant in the waiting list control group.

^l^ Assessment of the amount of total support received and satisfaction with support from family, friends, and the health care team (range, 4-20, with higher scores indicating more social support).^38^

^m^ One-month percentage-based rating of medication-taking behavior over the past month (self-rating question: “Over the past month, what percent of the time did you take all your diabetes medication as prescribed?”), with higher percentages indicating greater likelihood of taking medications as prescribed.^42,43^

^n^ Assessed by the revised Summary of Diabetes Self-Care Activities measure.^35^ Self-care activities reflect the number of days (of the last 7 days) the participant followed the diet plan, participated in physical activity, and tested blood glucose levels as recommended by the primary care physician.

^o^ Values are missing for 1 participant in the intervention group and 1 in the waiting list control group.

^p^ Assessed by the EuroQol 5-dimension visual analog scale (range, 0-100, with higher scores indicating better health [or best imaginable health state]).

## Discussion

This randomized clinical trial found that a clinical pharmacist and health coach–delivered mHealth intervention improved HbA_1c_ levels in African American and Latinx patients with T2D over 1 year compared with usual diabetes care. Importantly, improvements were maintained at 24 months. Based on these findings, this mHealth driven intervention may be considered an effective approach to improving blood glucose management in racial and ethnic minority patients with primary care access in urban environments.

This work built on a previous randomized clinical trial^9^ that included in-person clinical pharmacist support and tested the impact of adding a health coach. Results from that study showed an overall decrease in HbA_1c_ of 0.42 to 0.45 percentage points after 1 year, but the addition of a health coach did not impact this outcome as hypothesized.^9^ In contrast, the present study involving remote pharmacist support demonstrated an HbA_1c_ reduction of 0.79 percentage points. We attribute this reduction to addressing barriers (such as transportation) and the provision of mHealth tools (text messaging and remote iPad videoconferencing with cellular data). In the population studied, transportation to outpatient appointments is challenging,^46,47^ and telehealth occurs less frequently.^48,49^ Health coaches prepared patients for these remote encounters (eg, gathered glucose log and medication data) and reinforced pharmacist lifestyle and medication recommendations. Facilitating telehealth services may improve health disparities considering that broadband access and digital literacy vary across populations. Notably, during the COVID-19 pandemic, video telehealth was generally used less frequently among older adults, rural residents, and racial and ethnic minority patients.^50,51^

Our results aligned with those of previous studies^52,53^ that found short-term (<12 months) improvement in HbA_1c_ with clinical pharmacists providing medication management services in similar settings. The rationale for implementing an approach in which pharmacists, health coaches, and telehealth work together is compelling; there is growing evidence that team management of hypertension and hyperlipidemia, delivered remotely, is effective and provides outcomes comparable with in-person encounters.^24,54^ However, few randomized studies of medication management services have involved underserved communities and FQHCs, rigorously evaluated clinical outcomes,^8^ or had follow-up beyond 12 months.^55^ Additionally, future implementation studies are needed to better understand adoption and sustainability in larger networks of FQHCs with inclusion of economic outcomes.

Based on health coach activity logs, addressing social determinants of health (eg, food insecurity and language and literacy barriers) and providing social support were particularly valuable for pandemic isolation.^56^ The health coaches relayed patients’ social risks to pharmacists and primary care teams to help optimize care plans.^56^ Similar to other peer support studies, we observed improvements in perceived social support, which contributes to self-management activities and outcomes.^57,58,59^ Health coaches demonstrated potential in increasing technology use in diabetes management (eg, telehealth or, more recently, continuous glucose monitoring^60,61^). Additionally, we found durable improvement in HbA_1c_ at 24 months with extended self-management support. This finding was similar to the improvement observed in an 18-month trial including peer leaders who provided frequent contact for self-management support.^18^ However, challenges generally remain for health coach integration within health care organizations, including supervision and health record documentation.^62^ Furthermore, incorporating health coaches into the conventional health care system may modify their role as an independent community-based supporter to become a medicalized social service.^62^

### Strengths and Limitations

This study has several strengths. The study design measured 12 months beyond the intervention period for participants in the intervention group to evaluate the durability of HbA_1c_ change. The sequence used for the waiting list control group allowed additional patients to receive the intervention and enabled us to determine its impact beyond the experience of those who received the intervention in year 1. The study evaluated a practical approach to serving a diverse population with T2D and elevated HbA_1c_ who were at risk for complications^11^ despite having access to primary care. Additionally, we explored an innovative team model of health care using low-cost mHealth tools. The pharmacist and health coach team may be a worthwhile investment. For context, those with diabetes incur mean medical expenditures of approximately $16 750 per year, of which an estimated $9600 is attributed to diabetes.^63^

The study also has several limitations. First, because an academic medical center and affiliated network of FQHCs participated in the study, the results may not be generalizable to other environments, particularly nonacademic settings and those without clinical pharmacist or peer support services. Second, the intervention involved multiple components, including clinical pharmacists, health coaches, telehealth videoconferencing, and text messaging. It is impossible to determine their individual contributions to HbA_1c_ change, including through analyses exploring dose-response relationships. Furthermore, we are unable to identify a clear underlying mechanism to explain the improvements observed in HbA_1c_, notwithstanding unmeasured confounders. More complete secondary outcome assessments or additional assessments of prescription fills or electronic monitoring may have helped better understand the contribution of medication-taking behavior. We suspect that a more complex sequence of events and interactions are required for outcome change (ie, better glucose monitoring, medication reconciliation, adjustment of therapy, and adherence), and our study was not powered to detect more subtle independent changes in each of these domains. Third, despite the clinically significant improvement detected in HbA_1c_, most participants did not achieve their goal. Moreover, the waiting list control group experienced less intervention intensity due to COVID-19 restrictions on in-person contact, reducing potential impact on outcomes. Fourth, health coaches encountered challenges with engaging patients throughout a 1-year duration, which may have further limited their effectiveness. In some cases, health coaches’ difficulty in providing adequate needed resources can lead to patient feelings of hopelessness and diminished trust.^64^ Fifth, there is potential contamination between assigned groups. Clinics and PCPs likely encountered patients in both groups. Without clustering, the treatment contrast between groups may have been reduced, and the improvement in HbA_1c_ observed was possibly greater. Sixth, there was a substantial amount of missing data due to pandemic isolation for certain secondary outcomes (ie, body mass index and blood pressure). However, with the availability of home kits, adequate measurements of the primary outcome (HbA_1c_ level) were obtained.

## Conclusions

This randomized clinical trial found that clinical pharmacists and health coaches using mHealth tools improved HbA_1c_ levels among African American and Latinx adults with T2D and HbA_1c_ values of 8.0% or higher at baseline. Given their greater risk of diabetes complications compared with non-Latinx White adults, this strategy may be effective in reducing racial and ethnic disparities.

## Notes

Supplement 1.
Trial Protocol

Supplement 2. **eFigure.** Schematic Representation of Intervention Components **eTable 1.** Comparison of Characteristics Between Randomized vs Screened But Not Eligible/Not Randomized Individuals **eTable 2.** Comparison of Health Coach and Pharmacist Contact by Group **eTable 3.** Descriptive Statistics for Primary and Secondary Outcomes **eTable 4.** Summary of Clinician Encounters and Diabetes Medication Intensifications by Treatment Received **eTable 5.** Correlations Between Hemoglobin A_1c_ Change and Diabetes Self-Management Behaviors, Diabetes Medication Intensification, and Clinician Encounters During Intervention Year

Supplement 3.
Data Sharing Statement
