# Supplementary material for: Mobile Health Intervention in Patients With Type 2 Diabetes: A Randomized Clinical Trial
Source: JAMA Netw Open. 2023 Sep 29;6(9):e2333629. doi: 10.1001/jamanetworkopen.2023.33629 (PMC10543137; doi:10.1001/jamanetworkopen.2023.33629)
Supplement: Supplement 1. — Trial Protocol [file jamanetwopen-e2333629-s001.pdf]

Supplemental Online Content 1

mHealth for Diabetes Adherence Support Study Protocol

Principal Investigators:

Ben S. Gerber, MD, MPH
Professor of Medicine; Associate Chief, Division of Academic Internal Medicine and Geriatrics; College of Medicine; University of Illinois at Chicago
Institute for Health Research and Policy
Phone: 6-8872
Email: bgerber@uic.edu

Lisa K. Sharp, PhD
Associate Professor, Department of Pharmacy Systems, Outcomes, and Policy; College of Pharmacy, University of Illinois at Chicago
Phone: 5-3569
Email: sharpl@uic.edu

Co-Investigators:

Alana Biggers, MD, MPH. Assistant Professor of Clinical Medicine, Division of Academic Internal Medicine and Geriatrics; College of Medicine; University of Illinois at Chicago

Jessica Tilton, PharmD. Clinical Assistant Professor, Department of Pharmacy Practice; College of Pharmacy, University of Illinois at Chicago

Michael Berbaum, PhD. Director, Methodology Research Core, Institute for Health Research and Policy, University of Illinois at Chicago

Daniel Mihailescu, MD. Associate Professor, Department of Medicine; College of Medicine; University of Illinois at Chicago

Kevin Stroupe, PhD. Associate Professor, Stritch School of Medicine; Loyola University Chicago

Study Location(s): University of Illinois at Chicago, UI Health, Mile Square

Sponsor: National Institute of Health (NIH)
National Institute of Diabetes and Digestive and Kidney Diseases (NIDDK)

Version: 11.0

Date: 2/11/2021

TABLE OF CONTENTS

1.0 Project Summary/Abstract 3

1.1 Project Summary for the mDAS Sleep Sub-study Supplement 3

2.0 Background/Scientific Rationale 4

2.1 Background/Scientific Rationale for mDAS Sleep Sub-study Supplement 6

3.0 Objectives/Aims 8

3.1 Objectives/Aims for mDAS Sub-study Sleep Supplement 8

4.0 Eligibility 9

5.0 Subject Enrollment 10

5.1 Additional Subject Enrollment and Methods for mDAS Sub-study Sleep Supplement 11

6.0 Study Design and Procedures 12

7.0 Expected Risks/Benefits 23

8.0 Data Collection and Management Procedures 27

9.0 Data Analysis 31

10.0 Quality Control and Quality Assurance 31

11.0 Data and Safety Monitoring 31

12.0 Statistical Considerations 33

12.1 Additional Statistical Considerations for mDAS Sub-study 38

13.0 Regulatory Requirements 38

References 40

PHARMACY MANAGEMENT PROCOTOL 49

1.0 Project Summary/Abstract

Many African-Americans and Latinos with diabetes do not achieve recommended diabetes goals placing them at high risk for complications. Team-based models of care can help in reaching goals of therapy. Additionally, mobile health (mHealth) technologies can further improve outcomes among those more difficult to reach. This study will evaluate the impact of a team-based, mHealth intervention designed to improve medication adherence, healthy eating, and physical activity behaviors. We will compare this mHealth approach with usual care.

Clinical pharmacists and health coaches (HC) will deliver our proposed team-based intervention. mHealth delivery includes mobile phone text messaging, secure videoconferencing, and HC home visits. Pharmacists will focus on medication reconciliation and adherence. Health coaches will help identify psychosocial and environmental challenges to adherence in a culturally-sensitive manner. Together, they can assist in goal-setting, problem-solving, negotiation of competing priorities, and provide social support leveraging mHealth technologies.

Preliminary data from our research group supports the role of health coaches partnering with clinic-based pharmacists in improving diabetes outcomes in minorities. In the proposed mHealth intervention, patient-pharmacist videoconferencing will eliminate the need for in-person visits with a pharmacist, which is impractical for many low-income patients. In addition, our pilot work suggests that text messaging is a preferable means of communication and may facilitate more frequent contact with patients.

We propose a randomized, controlled trial to evaluate the effectiveness of an mHealth diabetes adherence support intervention delivered by clinical pharmacists and health coaches. We will randomize 220 patients through UI Health to either: (1) mHealth diabetes adherence support through clinical pharmacists and health coaches; or (2) usual care. After one year, patients completing the mHealth intervention will be monitored for an additional year while the usual care group receives the mHealth approach. Outcomes include medication adherence, hemoglobin A1c, blood pressure, and LDL-cholesterol levels. The specific aims include: (1) evaluate the effectiveness of an mHealth diabetes adherence support intervention delivered by clinical pharmacists and health coaches to African-American and Latino adults with uncontrolled type 2 diabetes; (2) evaluate the maintenance of improved diabetes behaviors as well as clinical outcomes one year after completing the intervention; (3) evaluate the cost and cost-effectiveness of mHealth diabetes adherence support compared to usual care; and (4) evaluate the reach, adoption, and implementation of mHealth diabetes adherence support based on the RE-AIM framework.

1.1 Project Summary for the mDAS Sleep Sub-study Supplement

As part of the parent R01 described above, Dr. Alana Biggers will conduct a smaller sub-study that is funded with a diversity supplement award to the R01. The award supports Dr Biggers gaining structured and unstructured experiences in research training over two years. With support of a mentored research study, she will investigate mobile health (mHealth) evaluation of sleep within the context of Type 2 diabetes. Sleep quality and duration are

modifiable risk factors that can help people improve diabetes control and reach diabetes goals. Although poorly understood, African-Americans and Latinos often report poorer sleep quality and/or duration and may be more susceptible to the metabolic effects of poor sleep. Additional research is needed to understand sleep behaviors and the possible impact on the health and well-being of these populations. Currently, sleep is rarely assessed by clinicians and behavioral interventionists. mHealth technologies can potentially improve the evaluation and study of sleep in these populations. This study will characterize sleep (quality, duration, and patterns) among African-Americans and Latino-Americans with uncontrolled diabetes, evaluate the effectiveness of mHealth in monitoring sleep and explore the relationships between sleep and diabetes outcomes. mHealth exploration includes mobile phone text messaging, wrist actigraphy use, and self-report surveys of sleep. Preliminary data from this study will inform the development of remotely-delivered approaches to sleep assessment and intervention (e.g., promotion of sleep hygiene). Initially, Dr. Biggers will lead a cross-sectional study of 40 participants to assess and explore the impact of sleep using mHealth.

The specific aims of this proposed study include: (1) explore subjective daily sleep quality, duration, and pattern of 40 inner-city African-American and Latino-American patients with poorly controlled type 2 diabetes (hemoglobin A1c>8%); (2) evaluate the feasibility and acceptability of objectively measuring sleep quality, duration, and patterns and sedentary behaviors via mHealth; and (3) explore the relationships between subjective and objective measures of sleep quality, duration, and patterns, as well as the relationship between sleep measures and glycemic control (i.e., hemoglobin A1c), quality of life, depression, and sedentary behaviors. Ultimately, this research may help demonstrate a potential role of mHealth evaluation of sleep, leading to innovative, behavioral strategies to improve diabetes-related outcomes.

2.0 Background/Scientific Rationale

Diabetes affects approximately 13% of African-American and Latino adults in the United States.[9] Aggressive management of blood glucose, blood pressure, and cholesterol through medication and lifestyle adherence are necessary to minimize adverse health outcomes.[17] However, barriers to adherence contribute to the fact that fewer than 20% of these ethnic minorities actually reach all therapeutic goals.[8,18-24] As a result, improvement in minority diabetes management remains a priority for research.[25]

Medical nutrition therapy, physical activity, and pharmacotherapy are essential components of diabetes self-management.[26] Adherence to diet and physical activity is low for a majority of Americans including those with type 2 diabetes.[27,28] Similarly, adherence to medication is poor:[29] 20-30% of prescriptions are never filled, and 50% of medications for chronic disease are not taken as prescribed.[30-32] Self-management among low-income, minority populations is often more problematic due to extensive barriers such as low health literacy, depression, lack of social support, poor patient-clinician communication, and limited access to healthcare.[16,20,21,33-39] Latinos may face additional barriers such as low English proficiency and other cultural issues. Low adherence may reduce treatment modifications and intensification contributing to poor diabetes control.[22,23] Systematic reviews of interventions designed to

improve medication adherence found few studies that improved clinical outcomes.[30,31,40,41] Sample sizes were small, and minorities (especially Latinos) were underrepresented. Further research is necessary to identify effective interventions that improve adherence in ethnic minority populations.

The *patient-centered medical home* (PCMH) approach has been endorsed widely[42,43] and involves diverse health professional teams working collaboratively with primary care physicians to provide high levels of care, access, communication, care coordination and integration.[42]

Clinical pharmacists improve glycemic control in diabetes management with their expertise in medication management and ability to adjust therapy in collaboration with providers.[44-49] Clinical pharmacists are widely accepted in this role; most state boards of pharmacy authorize collaborative drug therapy management protocols between clinical pharmacists and prescribers.[49,50] For example, Project IMPACT: Diabetes is a national research initiative supporting pharmacists working in community-based diabetes care teams, involving 25 underserved communities across the U.S.[4] Pharmacist-led interventions enhance adherence to chronic disease medication through patient education, use of adherence aids, and addressing medication-related issues (e.g., drug interactions and cost).[31,51-55] Our research with low-income minority patients suggests that in-person pharmacist visits are not feasible for many patients due to the added travel burden, cost, and time from work. Also, few studies of pharmacist-based models of care have included Federally Qualified Health Centers (FQHCs) or focused on low-income minority populations.[49]

Health coaches (HCs) are increasingly included as members of the medical home for vulnerable groups as their involvement contributes to improvement in diabetes self-management and A1c levels.[56-63] The Patient Protection and Affordable Care Act (ACA) formally recognizes the role of HCs in health promotion and education, disease management, patient navigation, improving health literacy, and advocacy.[64] UI Health has representation on the Illinois Department of Public Health (IDPH) Community Health Worker Advisory Board to support implementation across Illinois. Trained HCs are trusted by patients, understand socio-cultural barriers, and can help increase the relevancy of disease self-management to individuals who are struggling to manage competing priorities. Our research demonstrates that HCs can successfully collaborate with clinical pharmacists in addressing lifestyle and medication adherence.[16]

Mobile health (mHealth) includes “the use of mobile computing and communication technologies in health care and public health.”[65] Our mHealth approach includes tablet computer videoconferencing and cell phone text messaging components.

While the presence of mHealth applications is exploding in healthcare, few studies have explored the impact of videoconferencing on chronic disease management. Systematic reviews of Internet-mediated communication technologies fail to show clear effects on health due to variation in study design and technologies, heterogeneous study populations, and variability in contact frequency.[66-68] In diabetes care, technology-based interventions focus on transmission of blood glucose values with less attention on addressing barriers to self-management.[69] Empirical data suggest that videoconferencing in diabetes care is practical, potentially cost-effective, and reliable for disease management.[69,70] Video conferencing with clinical pharmacists to promote medication adherence is a growing model in rural practice (e.g., Department of Veterans Affairs[1] and Kaiser Permanente[2]) but not well studied, particularly with mobile devices

or involving urban, low-income minority patients.[71,72] Our study will focus on low-income African-Americans and Latinos and explore the use of videoconferencing between patients and clinical pharmacists, facilitated by HCs.

Text messaging interventions in diabetes management have shown mixed results and inclusion of low-income minorities has been low.[73-78] Few formal randomized controlled studies of text messaging exist.[79] One recent study showed short-term, modest improvement in cardiovascular risk factors in Australians with coronary heart disease.[80] While there are no clear, specific intervention components shown to improve outcomes, follow-up contact, individualized frequency, and tailored support likely improve acceptability and responsiveness by users.[81,82] Our study will include daily, tailored text messages with HC follow up to provide social support, improve self-efficacy, and adherence. Furthermore, the proposed study utilizes inexpensive technologies for text messaging and video conferencing, with greater potential for widespread adoption in underserved communities.

The widespread use of cell phones and text messaging among low income and minority populations has allowed HCs to increasingly adopt technology in their work.[83] To date, most of this research has been conducted outside of the U.S. utilizing technology for field-based data collection, sending reminders, providing health education, and communication.[13] Within the U.S., integration of low-cost technologies into community-based HC interventions remains an emerging area of research.[84]

Despite widespread use of mobile devices, little is known about the effectiveness of the technology to improve health care delivery or outcomes.[81] Systematic reviews show preliminary value of text messaging, patient education apps, and videoconferencing in chronic disease management, but most studies have been small in size, underpowered, low in quality, and include motivated, non-minority subjects.[65,69,70,78,79,85-88] Our work will provide needed evidence on the impact of mHealth diabetes adherence support delivered to low-income minority patients with uncontrolled diabetes.

2.1 Background/Scientific Rationale for mDAS Sleep Sub-study Supplement

Diabetes is a leading cause of death and disability in the US costing $45 billion in direct medical costs and reduced productivity. [6] Diabetes disproportionately affects minority populations [7] especially African-Americans and Latino-Americans. Studies have shown that lifestyle intervention can reverse and/or improve diabetes control. [8] While healthy eating and physical activity are well-studied behaviors in diabetes self-management, sleep is increasingly being recognized as a potential modifiable factor that can also lead to increased diabetes control. Studies have shown that people with short sleep duration, long sleep duration, or poor quality sleep have worse glycemic control, even without sleep-disordered breathing such as obstructive sleep apnea. [1,2] Poor sleep quality and duration can lead to weight gain and impaired glucose tolerance. [9–11] For these reasons, sleep is an essential lifestyle component to study in diabetes self-management.

It is well established that African-Americans, Latinos, and other ethnic and racial minorities are more likely to develop diabetes and develop complications from the disease. [7] It is possible that poor sleep duration and quality contribute to these health disparities. Data

exploring sleep quality within minority populations is very limited. However, existing data suggest that African-Americans are more likely to have worse sleep quality and shorter sleep duration compared to whites [4], whereas Latinos are more likely to have shorter sleep duration compared to whites. [4] Additional research is needed considering that these groups are also more likely to report psychosocial stress that contributes to inconsistent sleep patterns. [12,13] Furthermore, these populations are more likely to have nonstandard and non-day work hours (e.g., shift work) that contribute to inconsistent sleep and an increased risk of diabetes. [14–18] Further sleep research involving minority populations with diabetes is strongly justified and long overdue.

Despite evidence supporting a strong relationship between sleep and glycemic control, the mechanisms underlying the link are not well understood. Both diabetes and poor sleep are associated with more depression, lower quality of life, and more sedentary behavior. [19–26] In addition, socio-environmental factors secondary to poverty (e.g., neighborhood violence, social isolation, etc.) may result in higher levels of chronic stress that contribute to the link. The proposed study will explore these pathways by collecting detailed subjective and objective sleep assessments in patients with diabetes in addition to depression, quality of life, and physical activity (which are assessed in the parent grant). This study will begin to provide a nuanced understanding of the complex relationships between these highly relevant variables.

Little is known about the utility of mHealth to objectively evaluate sleep. The gold standard for studying sleep is polysomnography. [27,28] Unfortunately, this method is cumbersome and not feasible in a real-world setting. mHealth devices provide an opportunity to evaluate sleep in a natural setting. This is particularly valuable in mHealth intervention studies, including the parent study, where mobile assessments and interventions are incorporated. A few popular low-cost commercial activity trackers (e.g., Fitbit and Garmin) are widely available, however, they overestimate polysomonography total sleep time and sleep onset latency or underestimate wake after sleep onset. [29] Alternatively, wrist actigraphs represent evidence-based activity trackers that serve as comparable alternatives to polysomongraphy for measurement of sleep in the home. Actigraphs provide accurate measures of total sleep time, sleep percentage, movement, and wake after sleep onset. [27,28,30] In addition, their value may further improve with supplementary data from text message-prompted sleep diaries and selected questionnaires. [27] Together, the mHealth and traditional approaches can be used to evaluate home sleep environments and support efforts to develop sleep interventions.

The overall objectives of this supplement are to evaluate the feasibility and acceptability of mobile health approaches to assessing sleep duration and quality within minority populations known to have poor control of their type 2 diabetes. We will explore the relationships between sleep (duration, quality, and patterns), glycemic control, depression, quality of life, and sedentary behavior. Findings from this study will provide critical preliminary data to support Dr. Biggers' application for a career development award. Dr. Biggers' goal is to establish an on-going line of research as an independent investigator focused on developing sleep hygiene interventions to improve sleep duration and quality in minority populations with diabetes.

3.0 Objectives/Aims

We propose a randomized, controlled trial to evaluate the effectiveness of an mHealth Diabetes Adherence Support (*mDAS*) intervention delivered by clinical pharmacists and health coaches. This proposal is in response to NIH PA-14-334: Advancing Interventions to Improve Medication Adherence. We will randomize 220 patients through the University of Illinois Hospital & Health Sciences System (UI Health) to either: (1) mHealth diabetes adherence support (*mDAS*) through clinical pharmacists and HCs; or (2) usual care. After one year, patients completing the mHealth adherence intervention will be monitored for an additional year while the usual care group receives *mDAS*.

The specific aims include:

Aim 1: To evaluate the *effectiveness* of *mDAS* delivered by clinical pharmacists and HCs to African-American and Latino adults with uncontrolled type 2 diabetes. We will compare *mDAS* and usual care through the following outcome measures: hemoglobin A1c, blood pressure, LDL-cholesterol levels, and medication adherence.

Aim 2: To evaluate the *maintenance* of improved clinical outcomes and medication adherence one year after intervention completion.

Aim 3: To evaluate the cost and cost-effectiveness of *mDAS* compared to usual care.

Aim 4: To evaluate *reach*, *adoption*, and *implementation* of *mDAS* (RE-AIM framework)

3.1 Objectives/Aims for mDAS Sub-study Sleep Supplement

Sleep quality and duration are increasingly being recognized as modifiable risk factors for improving diabetes control. Studies have shown that short sleep duration (less than 7 hours), long sleep duration (more than 8 hours), and poor quality of sleep are associated with impaired glucose tolerance and insulin resistance. This increases the risk of developing diabetes and worsens glycemic control among those with type 2 diabetes. [1–3] Research specifically targeting sleep among low-income African-Americans and Latinos is lacking; however, available data suggest that these groups are more likely to experience problematic sleep patterns resulting in detrimental metabolic effects of poor sleep. [4] In addition, clinicians rarely assess sleep [5], though they are available to intervene on sleep hygiene. Collectively, these factors support the need to further investigate sleep in low-income minority populations with diabetes with intentions of developing remotely-delivered sleep assessment and intervention. The overall objectives of this study are to characterize sleep quality and duration among African-Americans and Latino-Americans with uncontrolled diabetes, evaluate the effectiveness of mHealth in monitoring sleep quality and duration, and explore the relationships between sleep and glycemic control.

The specific aims of this proposed study include:

Aim 1: To explore subjective daily sleep quality, duration, and patterns of 40 inner-city African-American and Latino-American patients with poorly controlled type 2 diabetes (hemoglobin A1c≥8%). Sleep quality, duration, and patterns will be measured by a validated sleep questionnaire, a mHealth version of a validated consensus sleep diary, and a home sleep environment questionnaire.

Aim 2: To evaluate the feasibility and acceptability of objectively measuring sleep quality, duration, and patterns and sedentary behaviors via mHealth. Study participants will wear an Actigraph Watch for seven days (including nights) and report their experiences with the device and assess its acceptability.

Aim 3: To explore the relationships between subjective and objective measures of sleep quality, duration, and patterns, as well as the relationships between the sleep measures, glycemic control (i.e., hemoglobin A1c), quality of life, depression, and sedentary behaviors.

Hypotheses 3a: Poorer sleep quality, duration, and patterns will be associated with higher hemoglobin A1c levels.

Hypotheses 3b: Poorer sleep quality, duration, and patterns will be associated with a lower quality of life, depression and more sedentary behaviors.

4.0 Eligibility

4.1 Inclusion Criteria

- Self-identified as Latino/Hispanic or African-American
- Verbal fluency in English or Spanish
- Latest A1c ≥ 8.0% (within 3 mo)
- History of Type 2 Diabetes (> 1 year)
- Between the ages of 21 and 75 years
- Unlimited mobile phone/text messaging plan with ability to reply to text messages
- Home environment capable of video conferencing with wireless signal per patient's report of cell phone reception in the home
- Receives primary care at clinical site for at least one year, with one visit during past year
- Able and willing to provide informed consent (agree to data collection requirements, accept randomization, agree to home visitation with HC and pharmacist involvement, and participate for two years)

4.2 Exclusion Criteria

- Unable to verbalize comprehension of study or impaired decision making
- Lives outside Chicago (3+ months/year)
- Family/household member already participating in same study
- Plans to move from the Chicago area within the next two years
- Pregnant or trying to get pregnant
- Currently receiving regular pharmacist support through Medication Therapy Management or equivalent
- Unable to send/read text messages on mobile phone
- History of, or planned, gastric bypass or transplant surgery
- History of bipolar or psychotic disorder
- Other severe comorbidities that require complex, aggressive, or palliative treatment, e.g., stage 4 or greater renal disease, liver failure, cancer (other than nonmelanoma skin cancer), terminal illness
- Investigator discretion for safety concerns

Supplement Sleep sub-study is offered only to English-speaking participants but no additional inclusion/exclusion apply.

5.0 Subject Enrollment

University of Illinois Hospital and Health Sciences System (UI Health) includes both inpatient and outpatient facilities serving a diverse population in Chicago. UI Health has 19 ambulatory centers staffed by Family Medicine and Internal Medicine PCPs and includes Mile Square FQHCs where care is provided independent of ability to pay. Four to six clinical sites will participate. All sites share access to the EMR, Cerner Powerchart, and employ clinical pharmacists through the UIC College of Pharmacy Practice.

We will employ the following recruitment/enrollment methods:

1. Passive Advertising: We will distribute study advertisement materials throughout UI Health, including clinical environments. Individuals will call research assistants (RAs) for eligibility assessment by phone.
2. Active Recruitment from Registries: The research assistants will work from a physician-approved list of patients with diabetes and A1c ≥ 8 identified from the electronic medical record. We will send letters informing patients that they may be eligible for the study and may opt-out of further contact. After one month, those who do not opt out will be contacted by the RAs to assess interest and eligibility.
3. Active Recruitment in Clinical Sites: On-site RAs will receive additional referrals directly from staff or patients interested in the study. In these situations, RAs will discuss the

study and assess eligibility in a private exam room within the clinic before or after a clinical encounter.

4. Diabetes Health Registry: RAs will call by phone individuals completing the Diabetes Health study (IRB 2011-0099) who indicated willingness to be contacted regarding future studies.

Chart review will be performed to establish eligibility (type 2 diabetes and A1c 8% or higher in past three months). An RA will complete an initial screening interview with the potential patient, either over the phone or in person in the participating clinics. RAs will ask screening questions (see Eligibility Screening attachment) and document the screening and eligibility (see Screening and Contact Information Sheet attachment). If the patient is eligible and interested, the RA will review the study in detail, using an IRB approved consent form as guidance. The RA will inform patients that the research is being conducted to improve medication management in diabetes care and to find out if pharmacists and health coaches help people reach goals of therapy. The RA will discuss the study protocol in detail as part of consent procedures and use the "teach back" method to ensure participant comprehension. The RA will clarify that the research procedures are separate from routine clinical care and participation is completely voluntary. All subjects must have a mobile phone with text messaging plan. Any individuals without an unlimited plan will not be reimbursed for text charges. The RA will confirm the patient's eligibility and willingness to meet with a health coach in their home. They will then obtain informed consent and HIPAA authorization. The patient will be offered a copy of the consent form for their own convenience.

Participants will then complete baseline data collection at the UIC Clinical Interface Core. All screening and study data will be recorded in REDCap. Screening reports will provide investigators with information regarding recruitment progress and reasons for ineligibility.

5.1 Additional Subject Enrollment and Methods for mDAS Sub-study Sleep Supplement

At the end of a regularly scheduled data collection point for the larger R01 study, English-speaking participants will be asked if they are interested in hearing the details of a sleep study (mHealth Supplement). Interested participants will be given a brief overview of the study and if they remain interested, at that time they will be fully consented for participation. If they are interested but unable to stay for the consenting process and/or additional data collection, another visit will be set up. Otherwise, after providing written informed consent, participants will complete a series of sleep related questionnaires that will be entered directly into REDCap. After completing the questionnaires, participants will be given a Philips Actiwatch with instructions for use. They will be asked to wear the watch for 7 consecutive days taking it off only if they are going to submerge it in water. They will also be reimbursed $10 for their additional time. Each day during the 7 day sleep period, they will answer daily questions through a survey about the previous night's sleep quality and process. The participants will have the option of a) completing the consensus sleep diary survey questions via text using the myTapp messaging system, or b) completing a web-based online sleep diary survey using a

secure application such as REDCap or Qualtrics. If the participants do not respond to the text message link, then research personnel will attempt to call the participant once to complete the day's sleep diary. At the end of the 9 days, participants will be contacted by telephone to complete a brief post-study assessment (see measures) and to schedule a day/time to return the actiwatch. Participants will be given the option of coming into the CRC to drop off the Actiwatch or they can have an RA come to their house to pick up the device. They will receive an additional $30 when the Actiwatch is returned. Recruitment goal is 40 individuals; although we expect to have to approach at least 60 in order to get 40 who will complete the study.

6.0 Study Design and Procedures

A randomized, controlled trial will evaluate the effectiveness of *mDAS* delivered by a clinical pharmacist and HC versus a usual care group. We will recruit 220 patients from UI Health. We recognize that all participants will have uncontrolled diabetes and require additional support from their healthcare team. Consequently, our usual care group will receive additional resources to meet basic standards of diabetes care and establish clinical equipoise between the two study groups. The usual care group will receive a referral to a diabetes educator and clinical pharmacist for medication reconciliation. These individuals are on staff and available to any patient, yet under-utilized. This group will receive a one-page contact sheet with the names and phone numbers of their local healthcare team as well as paper-based, low-literacy diabetes educational information. After one year, patients who have completed *mDAS* will be monitored for an additional year with usual care to evaluate maintenance, while the usual care patients receive *mDAS*. After recruitment, subjects will be randomized to receive either: (1) *mDAS* delivered by a clinical pharmacist and HC via home visits, videoconferencing, and text messaging; or (2) usual care. Study condition allocation will be completed after consent and baseline data collection. Using the REDCap Randomization Module, a random sequence of 220 subject assignments will be used. Blocked randomization (with varying block sizes) by clinical site, ethnic group (African-American and Latino), and gender will balance the proportion of participants.

6.1 Overview of Participant Procedures

<u>Recruitment and Randomization</u>

1. Potential participants will be identified by one of four recruitment methods previously described.
2. RAs will screen patients for eligibility and discuss the study protocol in detail. If the patient is deemed eligible to participate he/she will complete written informed consent and HIPAA authorization.

3. Enrolled participants will schedule an appointment at the UIC Clinical Interface Core (CIC) to complete baseline data collection, which includes a blood draw.
4. Participants randomized to *mDAS* will receive the intervention component in Year 1, while participants randomized to *Usual Care* will receive it in Year 2. RAs will inform participants of assignment.

Additional information for mDAS Sleep Sub-study Supplement

5. English-speaking participants will be asked to participate in a sleep sub-study to evaluate his or her sleep for 9 days at one of the regularly scheduled data collection follow up visits for the parent study.
6. RAs will screen willing participants for the sleep sub-study for eligibility and discuss the study protocol in detail. If the patient is interested, he/she will complete written informed consent for the sleep sub-study and complete the questionnaires. He/she will take an actiwatch to wear day and night for 7 days. Each day, they will receive mytapp messages asking about their sleep quality and process the night before. The Actiwatch will be returned at patient's convenience (drop it off or RA will pick it up). Reimbursement for the entire study is $40.

<u>Usual Care Component</u>

1. Participants will receive contact information for a diabetes educator and a clinical pharmacist for medication reconciliation.
2. Patients will receive a one-page contact sheet with the names and phone numbers of their local healthcare team as well as paper-based, low-literacy diabetes educational information.

mDAS - <u>Pharmacist Component</u>

1. Pharmacists will provide medication and disease management services to patients by following a Pharmacist Management Protocol (see attached).
2. The initial pharmacist videoconference will include medication reconciliation and review therapeutic goals.
3. Follow up videoconferences will occur every other month. The interval duration may increase/decrease based on individual preference and when subjects reach goals.
4. These subsequent visits will involve an evaluation of adherence, medication reconciliation, and a review of home glucose and/or blood pressure monitoring data, an evaluation of drug interactions and side effects, goal setting, basic lifestyle

modifications, pillbox use, development of a low-literacy medication list, and other adherence aids.

5. Pharmacists will adjust therapy according to the plan of care under PCP guidance and notify PCPs of agreed upon modifications through EMR notifications. Side effects will be conveyed to the PCP (if the PCP is not available, the covering physician will be contacted as per Pharmacist management Protocol).
6. Pharmacists will forward all electronic progress notes to PCP EMR "Inbox" after each encounter.
7. If the PCP feels that the patient should not remain in the study the provider can inform the Co-PIs to disenroll the participant.

mDAS - Health Coach Component

1. All health coaches will receive standardized training/re-training via two educational curricula: (1) the *Diabetes Education Empowerment Program*; (2) Dr. Bodenheimer's *Training Curriculum for Health Coaches*; and (3) PEARLS for problem solving in depression management.
2. The coaches will communicate with their patients through home visits, phone calls, clinic encounters, and text messaging. Coaches will use *mytapp* to schedule text messages (messages will be tailored to individual and reflect patient goals).
3. Coaches may evaluate food inventory, medication adherence, and insulin injection techniques. Coaches will check glucose levels and blood pressures as needed.
4. Coaches may show Living Well with Diabetes lessons via iPad for education.
5. Coaches will participate in goal setting procedures. They will use the Purple Binder to access community resources.
6. Coaches will facilitate videoconferencing with patients. A multidisciplinary meeting will include coaches and pharmacists to communicate on a weekly basis about active participant issues.
7. Coach activity will be collected on a standardized electronic form completed after every participant contact. Coaches will document contact in the EMR.

Crossover Transition

1. One year after enrollment, the participants receiving *mDAS* will have the intervention components phased out to assess maintenance. The participants in the usual care group during year 1 will receive *mDAS* support for the subsequent year to assess intensification.
2. Coaches will help participants enlist social support and community resources for long term assistance with medications.

3. Coaches may periodically be contacted by disengaged participants during the following year. These communications will be logged.

Retention

1. Monetary reimbursement for each data collection
2. Periodic phone calls to verify address and phone data
3. Use of secondary alternative contact information
4. Any "lost" individuals will be searched for using a comprehensive standardized web based search protocol which included:
    a. Five people locators
    b. Major social networking sites, specifically those with more minority involvement
    c. Department of corrections database
    d. These searches will be conducted on the target individual as well as secondary contacts
    e. All potential contact will be private. No study related information will be conveyed in public through these searches.

Health Coach Fidelity

1. To continuously evaluate the fidelity of the pharmacist and health coach components of the intervention, investigators will review health coach and patient medical records of 10% of participants randomly selected.
2. In addition, we will monitor health coach activities through encounter forms that include participant interactions and duration of time spent on activities.
3. Drs. Gerber and Sharp will attend multidisciplinary weekly meetings with health coaches and pharmacists and review active patient issues.

Data Collection

1. This research will be performed at the CCTS Clinical Interface Core.
2. Surveys will be conducted by interview format. This information is only being collected for research and will not be shared with others. This will take approximately 60 minutes.
3. We will draw some blood from each subject's arm to measure Hemoglobin A1c and cholesterol profile. Each subject will be asked to fast (may drink water but may not eat food for 12 hours prior to the test). The amount of blood drawn will be about 6 ml (approximately one teaspoon).

a. Briefly, a total of 6 ml of whole blood will be collected in one gold SST top vacutainer tube and one lavender EDTA tube on each of the blood draws.
b. Once collected the blood will be delivered to the Alverno lab for processing.

4. Each subject's height, weight, and blood pressure will be measured, similar to how this is done in the doctor's office.
5. The subject will provide contact information for all of the pharmacies where prescriptions have been filled for the past year. The subject will allow study personnel to contact these pharmacies to collect information on the type, number, and dates of prescriptions filled.
6. Each subject will be asked to return after 6, 12, 18, and 24 months for similar testing (surveys, blood tests, height, weight, blood pressure).

Additional Data Collection for mDAS Sub-study Supplement

7. Each subject will be asked to complete surveys in interview format on personal sleep practices. The surveys include: Pittsburgh Sleep Quality Index, PROMIS Sleep-related Impairment Short Form 8a (8 items) and PROMIS Sleep Disturbance Short Form 8b (8 items), an assessment of sleep apnea and restless leg syndrome. This will take approximately 20 minutes. Patients will be compensated $10 for this initial sleep sub-study data collection.
8. The RA will provide instructional tutorial on using the wrist actigraphy (Actiwatch) to the participants. This will take approximately 5 minutes
9. Study participants will wear a wrist actigraphy for 7 days and 7 nights and answer custom questions on sleep after the participants wake up. The participant has the option of answering the questions via myTapp messages or through a web-based online sleep diary survey using a secure application such as REDCap or Qualtrics. These questions will be modified from a validated consensus sleep diary.
10. At the end of the 9 days, participants will be telephoned to complete a post-study evaluation and to schedule a day/time to return the actigraph watch.
11. Participants will be compensated $30 for sub-study completion by research personnel.

On the consent form, participants will be asked for permission to use the data collected within the larger mDAS study as part of the analyses in this sub-study. Patients are allowed to decline and still participate in this portion of the research.

<u>Temporary modifications to data collection during COVID-19 pandemic</u>

Due to the COVID-19 pandemic and social distancing orders, the following modifications will be made until research operations allow adequate in-person contact.

1. Data collection surveys as described above will be conducted by telephone with the possibility of using Doximity (HIPAA secured videohealth) in some occasions.
2. Subjects will be compensated for data collection visits with a gift card instead of cash. Gift cards will be purchased online from major online retailers and mailed directly to subjects.
3. Subjects will be offered the option to complete the Hemoglobin A1c test and Cholesterol panel at home using FDA-approved home test kits from Home Access Health Corporation. Each test requires a finger stick using a lancet (provided) to produce 5 drops of blood for the sample. The total time required to complete the tests is 20-30 minutes. The following steps will be taken to inform subjects about the kits and obtain results remotely.
    a. Data collectors will call subjects to provide details about the kits and determine if they are willing and able to complete the lab tests at home.
    b. If so, coordinator will prepare one of each kit to be mailed to the subject. Coordinator will sanitize work surfaces thoroughly and wear gloves and a mask while packaging any kits.
    c. Each kit has an identification number which will be linked to the subject's study ID. Home Access also requires a date of birth (DOB) to identify each sample. To protect the subject's PHI, we will create a master list with shifted DOBs for subjects using the REDCap date shifting feature. With this feature, REDCap randomly shifts dates up to 364 days upon downloading the data. The shifted DOB and the study ID will be used to label the kits.
    d. A list of assigned kits with shifted DOBs and study IDs will be sent to Home Access via a secure FTP (sFTP) server. No PHI will be sent to Home Access.
    e. The subjects will complete the tests with guidance from the data collector (over the phone or Doximity) and mail them to the lab using the prepaid envelope provided by Home Access.
    f. Samples will be processed by Home Access and results will be sent daily to coordinator via sFTP server.
    g. A $20 gift card for completing the home test kits will be sent to subject once a result is confirmed.

    Note that all subjects will have the option to decline home testing and wait until research operations resume and they can complete the blood draw per the usual protocol. Subjects who choose to wait will also be compensated $20 for completing the blood draw *only* for survey data collection visits completed during the COVID-19 pandemic. Once research operations

resume, subjects will be paid as outlined in section *6.2 Transition and Retention.*

Evaluation of Pharmacist Activity and Medication Changes

1. Chart review by a research assistant will define the number of PCP and pharmacist encounters as well as the number of pharmacist- or physician-initiated medication changes.

6.4 **Components of *mDAS***

Videoconferences. Pharmacist videoconferences 30-45 minutes in duration will be scheduled every other month or as needed. HCs will reserve videoconference appointments using an online system with dedicated days/times pharmacists are available. All patient encounters with pharmacists are remote via videoconferencing with HCs present in the home using VSee. Vsee transmits personal health information securely, is low cost, and is available for use on desktop computers and mobile devices (e.g., iPads). Similar to Skype, Vsee provides real-time person-to-person audio and video communications. However, it is most suitable for health-related communication, as it is FDA registered and HIPAA compliant (using 256-bit AES, Advanced Encryption Standard). Vsee uses “peer-to-peer” sessions so information is not stored on a server.

Videoconferencing will follow the American Telemedicine Association practice guidelines: (1) pharmacist and patient/HC identity verification; (2) informed consent; (3) physical environment (privacy, lighting, noise); (4) education and training (pharmacist and HC); (5) alternate communication (e.g., telephone contact in case of disruption of service); and (6) documentation in UI Health EMR (by pharmacist). All study pharmacists and HCs will receive standardized training on use of videoconferencing which includes scheduling, preparing the environment (home or pharmacist office), patient education on telehealth, and documentation. We will evaluate HC and pharmacist competency. All encounters will have contingency plans in place for technology problems (e.g., inadequate signal for video streaming), including use of mobile phones for all HCs. Videoconference content includes standardized medication and disease management.

Pharmacist Services. Pharmacist services follow a standardized Pharmacist Management Protocol. All participating clinical sites have clinical pharmacists providing in-person disease management services and follow a similar protocol. All participating pharmacists have faculty appointments in the UIC Department of Pharmacy Practice. The UI Health system is a teaching hospital and as such, pharmacists at participating sites train an ongoing rotation of pharmacy residents and students. With the patient’s permission, residents and students may observe clinical consultations performed by the pharmacist. Residents and students will not provide any clinical care to patients and will not perform any videoconference visits. They will not have any role in this study. The clinical pharmacist reconciles medications during the first encounter with

assistance from the HC in the patient's home. Follow-up *mDAS* pharmacist activities include evaluation of medication and lifestyle adherence, reviewing home glucose and/or blood pressure monitoring log data, identifying therapeutic goals, formulating an approved plan of care, assessing for changes in medications, and documenting the plan in the EMR.

In addition, pharmacists provide education related to medication (name and purpose of medications; time, strength, and method of administration); drug interactions and side effects; goals of therapy; basic lifestyle modifications; and use of pillbox, low-literacy visual medication lists, or other adherence aids. Pharmacists educate and encourage lifestyle changes based upon ADA nutrition and physical activity guidelines. They propose medication changes based on algorithms and protocols derived from national guidelines under physician guidance. Pharmacists will have access to home glucose monitor readings during the videoconference, as well as ambulatory blood pressures obtained by the HC during home visits. Pharmacists will set the goals for A1c and blood pressure collaboratively with PCPs (e.g., A1c ≤ 7%) as is the case in regular clinical practice within our setting. These procedures are currently followed with clinical pharmacists in ambulatory management of complex patients with uncontrolled diabetes.

Overall, there is mixed evidence regarding the benefits of aggressive glycemic control. In the proposed study, PCPs and pharmacists will adopt the ADA approach to individualized care, where the general goal for non-pregnant adults is A1c less than 7%. They may decide upon less stringent goals for those with a history of severe hypoglycemia, limited life expectancy, advanced complications, or extensive comorbid conditions. Pharmacists routinely monitor hypoglycemic events, address prevention and review treatment. This includes three steps: (1) addressing hypoglycemia with every patient contact; (2) applying principles of appropriate therapy (education, empowerment, frequent glucose self-monitoring, flexible medication regimen, individualized goals, professional guidance); and (3) considering risk factors for hypoglycemia. In addition, pharmacists will follow the 2013 American College of Cardiology/American Heart Association guidelines for lipid management (e.g., calculating 10 year atherosclerotic cardiovascular risk to determine statin intensity).

PCPs and pharmacists communicate routinely regarding patient care and are located in the same area within the medical setting. Pharmacists review EMRs including blood test results, clinical progress notes, problem and medications lists, drug allergies, hospitalization records, and emergency room reports. Non-urgent communication and electronic progress notes from each pharmacist encounter will be sent to the PCP through inbox messaging and note forwarding within the EMR. Pharmacist progress notes include a detailed list of medications, estimated adherence levels, and home glucose/blood pressure monitoring log information.

Health Coaches. The study includes both African-American and bilingual/bicultural Latino health coaches representative of the communities they serve. All of our current HCs are the first person in their families to earn an undergraduate college degree, come from low-income backgrounds, and recognize the importance of cultural-based norms. All HCs have an undergraduate degree in or related to Community Health with experience conducting home visits. They receive 80 hours of standardized training/retraining beginning with diverse Latino and African-American cultures in America and the influence of poverty upon health. HCs provide culturally sensitive support and tailor based on participant personal preferences. Fundamental education in diabetes includes the Diabetes Education Empowerment Program

(DEEP) targeting literacy and cultural awareness and Dr. Thomas Bodenheimer's Training Curriculum for Health Coaches. HCs shadow a clinical pharmacist to understand medication use, adherence, glucose and blood pressure monitoring, insulin injection, and medication reconciliation. On-going training is provided every two months on related topics that reinforce and expand the initial training. We provide training in videoconferencing (e.g., scheduling and practice encounters), text messaging (e.g., text application practice and standardized safety procedures), and conducting home visits in a culturally sensitive manner. HCs receive routine orientation to clinical operations and staff at their primary care locations. Standardized safety procedures related to text messaging and home visits are also addressed. Dr. Colleen Fairbanks, a consultant, provides training in motivational interviewing related to adherence. To evaluate clinical skills, the HCs will demonstrate reliable measurement of blood sugar, blood pressure, pulse, and administration of insulin (though HCs do not administer insulin to patients).

The HC component involves monthly home visits with on-going telephone support. Every other month a visit will include facilitation of the pharmacist videoconference, with the patient spending an additional time with the HC afterwards for reinforcement. HC visits including pharmacist videoconferencing will last 60-90 minutes total, while the HC visits without pharmacist involvement will last 30-45 minutes.

The HCs work with the pharmacist to evaluate adherence (e.g., check fill and expiration dates), assist in medication reconciliation, review home glucose and/or blood pressure monitoring data, and provide reinforcement of proper medication use. Finally, HCs debrief patients after pharmacist encounters to reinforce and clarify recommendations and plans. HCs and pharmacists will communicate with each other between videoconference encounters by phone or secure e-mail as needed to coordinate efforts. HCs provide pharmacists with valuable insights into culturally-influenced lifestyle issues and health beliefs.

Home visits include the HC, patient, and family members or co-residents as appropriate and permitted by the patient. This is particularly helpful when someone else prepares meals for the patient. HCs address nine critical content areas over the course of the one-year intervention: relationship building, diabetes education, medication reconciliation, adherence barriers, engaging social support, navigating the healthcare system, exploring emotions, movement or physical activity, and nutrition. HCs evaluate key points in each content area which guides their work with each patient. HCs can check blood pressure and blood sugar to share with the pharmacist and other providers. HCs will document summaries of all patient communication in the EMR through remote access and forward electronically to PCPs and pharmacists.

HCs will attend to lifestyle and medication adherence issues by identifying and tracking barriers, problem solving, goal-setting, and providing supportive accountability. Using motivational interviewing techniques, HCs help patients identify diabetes-related goals and create action plans. Many of our patients are socially isolated. An important HC role is to identify extended family, friends, or neighbors for support. HCs engage support for tangible tasks tailored to the patient and directed at positively impacting adherence.

Diabetes education is facilitated by our culturally appropriate multimedia education iBook, Living Well with Diabetes/Viviendo Bien con Diabetes (LWD). This iBook is available in English and Spanish and targets African-Americans and Latinos through video testimonials, food choices, and various lifestyle issues. HCs present specific chapters in LWD on a tablet computer

along with other visual, low-literacy materials to reinforce specific diabetes self-management concepts. In addition, HCs can utilize available evidence-based tablet computer tools in decision support. HCs are trained on diabetic medical nutrition therapy and basic physical activity recommendations for those with poor mobility so that they can work with patients to set individual goals. Education will address realistic and achievable food choices, portion sizes, cooking preparation, relationships between medications-meals-glucose levels, and integration of movement into lifestyle. HCs routinely identify basic resource needs that they address by facilitating connections with food pantries, free park district activities, or assistance paying utilities. Additional relevant resources are identified using online databases, such as purplebinder.com and healtherx.org.

Text Messaging. All text messages will be tailored by health coaches and sent through a custom software application (*mytapp*). Dr. Gerber developed *mytapp* for community-based health behavior research. *Mytapp* sends messages immediately or at a scheduled date/time, recurrent messages (daily, weekly, or weekdays), group messages, or multiple question surveys. *mytapp* requires a password for access and is restricted to trained research personnel. HCs receive training on *mytapp* and schedule messages for their patients regarding appointments, medications, and goals. The content of text messages will parallel our conceptual framework providing social support, competence, perceived barriers and benefits of treatment, and severity and susceptibility of complications. Health coaches will monitor their own patients' responses for their cohort. Additionally, the project coordinator will monitor all *mytapp* activities including subject responses.

Goals set jointly by HC and patients are SMART (specific, measurable, achievable, relevant, and time-specific). Action plans are culturally tailored to subject individual needs, preferences, and resources. African-American HCs will highlight specific activities and resources in African-American communities. Likewise, Latino HCs include Spanish text messages (if Spanish is the preferred language) and different community-based resources and educational materials. Activity choices may include routine medication use, food choices, physical activity, stress reduction, smoking cessation, or other behavioral targets. Subjects indicate their level of confidence in success to help refine the frequency and intensity of activity though motivational interviewing techniques. HCs will help their patients anticipate barriers, discover potential solutions, and establish follow up plans. They employ problem-solving strategies and engage community resources as needed.

Text messaging will further enhance HCs' ability to support their patients. They will direct messages to maintain motivation, elicit feedback on progress, and screen for barriers that may reduce chance of success. For example, a morning text message may ask a patient how they did on their goal the previous day. Responses may be "yes/no", a rating scale, or other measure of activity. The subject will receive an automated response based on progress made, and additional messages (tips/problem solving messages) if not achieving goals. No more than seven messages will be delivered weekly, except for optional medication reminders.

Example Text Message Templates

Message Type	Frequency	Example
Medication Reminder	1-4 times/day	"Hi, Ms. Brown, just checking to see if you took your morning pills."

Refill Reminder	Monthly	“Make sure you don't run out. Check to see when a pill refill is due.”
Appointment Reminder	Variable	“Just a reminder that you have a doc’s appointment today.”
Goal Monitoring	Variable	“Remember your goal. Did you take your meds last night?”
Glucose Monitoring	Variable	“Hello, Mr. Marquez, have you had time to check your sugar today?”
Self-Efficacy	Weekly	“Taking your meds is within your control. You can do it.”
Motivation	Weekly	“You have come a long way! Keep up the good work!”

Temporary modifications to data collection during COVID-19 pandemic

Due to the COVID-19 pandemic and social distancing orders, all pharmacist and health coach contact with participants will be made remotely and not in-person. This includes phone calls and Doximity video telehealth.

Additional text messaging for mDAS Sub-study Supplement

Participants in the sub-study will be asked to answer daily questions on their previous night’s sleep. They will complete the seven day sleep assessment surveys based on their preference by either text message surveys using myTapp or they will receive a text link to an online-based survey through a secure, web-based data collection application, such as REDCap or Qualtrics. The text messages will serve as a modified mobile consensus sleep diary for patients during the sleep period. The questions will include the following:

1) What time did you get into bed last night?
2) What time did you try to go to sleep?
3) How long did it take you to fall asleep?
4) How many times did you wake up, not counting your final awakening?
5) In total, how long did these awakenings last?
6) What time was your final awakening?
7) What time did you get out of bed for the day?
8) How would you rate the quality of your sleep? a) very poor, b) poor, c) fair, d) good, or e) very good? Please text the letter that corresponds to you (if using text message response).
9) In total, how long did you nap or doze?
10) Comments

Thanks for your participation.

Transition and Retention

Despite having "access" to healthcare, participants will be economically vulnerable individuals with uncontrolled diabetes. Ethically, we acknowledge that the "usual care" currently received often does not meet patients' needs or standards of care. Therefore, participants assigned to usual care will receive initial support to engage existing resources available through UI Health, such that healthcare providers will consider this an acceptable alternative upon randomization (clinical equipoise).

Upon randomization, participants will receive referrals to a diabetes educator and clinical pharmacist for medication reconciliation, adherence support, and education. Staff can schedule the visit at data collection. This is important as our prior work with medication reconciliation identified discrepancies in almost 60% of patients. A one-page list of clinic resources with names and direct telephone numbers (e.g., social worker) will be provided so they can more easily navigate the system. Finally, low-literacy, paper-based diabetes education information will be provided. At the end of year 1, these patients will cross-over and receive *mDAS*. Prior to this transition, HCs will work with the patients and their social support network to prepare them for the transition. Participants randomized to usual care will receive *mDAS* for one year.

Retention strategies. Participants will be reimbursed $30 for each data collection. Participants assigned to the intervention in year one will receive an additional $50 at 6 months and 12 months if they complete at least one videoconference before each data collection. Participants assigned to the intervention in year two will receive an additional $50 at 18 months and 24 months if they complete at least one videoconference before each data collection. Participants who complete all five data collection visits and videoconferences with the pharmacist as described above will receive up to $250 for completing the study.

Additional Retention Strategies for mDAS Sleep Sub-study Supplement Participants will be reimbursed an additional $40for participating in the sleep sub-study and turning in the wrist actigraph.

Temporary modifications to compensation during the COVID-19 pandemic. Participants may choose to be paid through their preferred electronic pay app (such as QuickPay/Zelle, Venmo, or Paypal) OR a gift card of their choice from a list of online retailers such as Walmart, Target, Amazon, or Visa. Gift cards will be issued in the amounts as described above for each data collection visit and can be mailed, emailed or texted depending on the vendor.

Due to social distancing orders, participants will have the option to complete their HbA1c and cholesterol profiles using a home test kit. They may also choose to wait and come in to the CRC for a blood draw once research operations resume. Because of the added time and effort required for each of these options, an additional $20 will be given to subjects for lab tests completed during the COVID-19 pandemic only. This additional incentive will not be offered once in-person interviews and phlebotomy resume.

Other retention strategies include: (1) periodic phone calls/ mailings to verify contact information; and (2) use of alternative contact information. We have developed a comprehensive standardized web-based search protocol that includes five people locators, major social networking sites including those with more minority involvement, and the department of corrections database. Searches are conducted on the target individual as well as alternative contacts.

7.0 Expected Risks/Benefits

<u>Risks and Protections</u>

The main risks include: (1) a small risk that subjects will have blood sugar or blood pressure levels that are too low or too high; (2) a small risk that the confidentiality of the subjects' records will be breached; (3) a small risk that subjects will feel annoyed by receiving study text messages; and (4) a small risk that a subject will experience pain or bruising at the site where blood is drawn for phlebotomy (less common risks include fainting, the formation of a small blood clot or swelling of the vein and surrounding tissue, bleeding from the site, and infection). It is unlikely that any of these risks would result in serious consequences. For all patients, an alternative to enrolling in the study is not participating (patients may continue usual health care services at UI Health outside of the study, including diabetes education).

Additional risks for those enrolled in the sub-study include a small risk the patients may develop a rash, irritation, or discomfort from wearing a wrist actigraph for 7 days/nights.

HIPAA regulations focus more on minimizing and managing risk rather than the actual information technology utilized. We will utilize a comprehensive approach to maximize data security for videoconferencing and text messaging services. Protected health information (PHI) will be exchanged through videoconferencing but not in text messaging. We will include an information privacy and security committee to advise, assist, and monitor our research policies using technology and for documentation. The committee will include an IT security expert, privacy official, and a clinician from UI Health. The committee will: (1) conduct a formal risk analysis; (2) develop a risk management strategy based on risk analysis; (3) implement strategy and train staff; and (4) monitor risk continuously. Text messages will have the health coach name pre-pended to messages to remind participants they come from health coaches (e.g., “Monique: Just a friendly reminder – it’s time for your meds, Carol.”). Patients will be reminded that text messages are sent in an automated fashion by computer. Thus, any replies may not be read immediately. Text messaging by patients should not be considered an Emergency Response System. Furthermore, they will be reminded that urgent health questions should be directed to their physician and not sent in a text message. HCs will monitor daily message feeds containing routine responses from patients.

Extreme Blood Sugar or Blood Pressure Levels: If a patient takes too much or too little medication, blood sugar and blood pressure levels may be too low or too high. The expected frequency of experienced side effects among participants is comparable as those receiving usual care taking the same medication. However, greater intensification of therapy could potentially increase this risk. To minimize risk, pharmacists and PCPs follow standard algorithms for glycemic and blood pressure management (i.e., American Diabetes Association and Joint National Committee on Prevention, Detection, Evaluation, and Treatment of High Blood Pressure) and use standard precautions in adjusting therapy: (1) addressing side effects (e.g., hypoglycemia) during every patient contact; (2) applying principles of aggressive therapy (education, empowerment, self- monitoring, flexible medication regimen, individualized goals, professional guidance); and (3) considering risk factors for hypoglycemia or hypotension. HCs also receive specific training on how to respond to extreme blood sugars and blood pressures if this occurs during a visit. They will be trained to respond in manner that is appropriate for their positions as trained lay-people.

Currently, at least one-third to two-thirds of patients with diabetes do not take their diabetes and hypertension medications correctly. In contrast, study participants will receive greater monitoring and support than other patients receiving usual care, which may help them

take medication more properly. The study procedures will allow for medication adjustment in a logical, conservative fashion, whereas many patients may adjust therapies on their own, or with infrequent or ineffective provider contact, not adjust at all. All pharmacists and health coaches receive extensive training and monitoring to deliver appropriate assistance to patients. We have implemented additional protection with PCP (or covering physician) contact if necessary at any time, day or night. Based on the frequent contact and monitoring involved, participants have less extreme blood sugar or blood pressure levels than those receiving usual care.

Poor Information: It is possible that subjects may receive and act on inaccurate information or misinterpret accurate information given the team approach and novel communication methods utilized. However, it remains more likely that there is a greater risk of misinformation from providers when additional support is not available, especially due to language and cultural barriers. To address this, HCs receive training and ongoing evaluation to reduce the risk of providing misinformation. HCs serving Latino patients will be bilingual, and all communication as well as informational materials will be in either English or Spanish to avoid a potential language barrier. In addition, HCs work closely with pharmacists and communicate regularly, providing a means to obtain accurate information and receive prompt and clear answers to their questions. To address any possibility for subject concern regarding information received, we will encourage them to seek help from their primary care physician (PCP) and pharmacist if they are uncomfortable with any of the information provided by health coaches.

Breach of Confidentiality: There is a small risk of breach of confidentiality, regarding health information and collected research data. All research staff, including pharmacists and HCs, will have formal IRB human subject protection and HIPAA training. Additional HC training includes discussions on maintaining confidentiality of health information, including communication with health providers. For data collection, all subjects will be assigned a blind code number. These lists will be kept in a locked file and will not be shown to staff. Data collected from computer use files will have the name removed and the code number attached by the research coordinator. Project staff who have access to the data will not have access to subject names. No clinical staff who have direct contact with the subjects will have access to research data. Finally, there will be no personal information stored on the server itself; and all e-mail communication will be encrypted and secure.

Videoconferences will be conducted through VSee. VSee provides real-time person-to-person audio and video communications. It is most suitable for health-related communication, as it is FDA registered and HIPAA compliant. VSee uses 256-bit AES (Advanced Encryption Standard) and "peer-to-peer" sessions so information is not stored on a VSee server. Although VSee allows for local file storage which may be considered electronic personal health information (e-PHI), UI Health does not currently have electronic storage of such communications into the electronic medical record. Pharmacist notes abstracting the relevant details of the videoconference encounter will be recorded in the medical records. Overall, the risk of accidental disclosure of e-PHI is very small.

Text messages will not include any identifying information nor will any personal health information be disclosed by our team. Patients will be instructed not to use text messaging to share health information. Patients will be reminded that text messages are sent in an automated fashion by computer. Thus, any replies may not be read in a timely fashion. Text

messaging by patients should not be considered an Emergency Response System. Furthermore, they will be told and reminded that urgent health questions should be directed to their physician and not sent in a text message. If an individual does share e-PHI through text messaging, the information will be permanently deleted and the patient will be educated regarding this process.

Feeling annoyed by text messages: Subjects will receive regular scheduled text messages during the study. Participants who no longer wish to receive reminders or text messages may reply with “STOP.” Twilio automatically will block *mytapp* from sending messages if this is received; thus, messages will be immediately discontinued without requiring contact with research team. However, patients have the option to re-subscribe with “START” again and resume messages. *mytapp* also color-codes blocked messages as “red” which will be easily detected by health coaches. This allows the HCs to identify problems including loss of mobile service.

Phlebotomy: Subjects may have pain or bruising at the site where blood is drawn for phlebotomy. Less common risks include fainting, the formation of a small blood clot or swelling of the vein and surrounding tissue, bleeding from the site, and infection. The Clinical Interface Core (CIC) employs health professionals for all phlebotomy services.*Home test kits*: Subjects may experience very brief discomfort after the finger stick and from squeezing drops of blood into the sample well. This will be comparable to the pain they experience when they undergo glucose testing. Alcohol pads, gauze and bandaids are provided with each kit to keep the site clean. Tips for minimizing discomfort and increase blood flow are included in the kit and instructional video online.

While unlikely, there is a slight risk that a participant may indicate thoughts of suicide or self-harm by scoring a 2 or 3 on item 9 of the PHQ-9 questionnaire. If this occurs, the data collector will contact Dr. Lisa Sharp on her cell phone immediately upon completion of the interview or during a break in the interview (if appropriate). Dr. Sharp is a trained clinical psychologist and as such will come to the CIC, meet with the patient and evaluate if further action is needed to ensure the participant’s safety. If Dr. Sharp is unable to be reached, Dr. Gerber will be paged or reached by cell phone. Dr. Sharp or Gerber will assess the participant including suicidal ideation, intent, and plan. Then, Dr. Sharp or Gerber will intervene appropriately, which may include providing resources for mental health support, facilitating referral to psychiatry, or immediately escorting the participant to the UI Health emergency room if urgent evaluation/intervention is needed to ensure personal safety, security, and stability.

<u>Benefits</u>

This study may provide additional, generalizable knowledge regarding the impact of the mHealth diabetes adherence support intervention, including HCs and clinical pharmacists in helping minority groups with uncontrolled diabetes. The expectations are that all subjects may potentially benefit from the support provided. In the United States, underserved African-American and Latino populations are disproportionately affected by type 2 diabetes and face greater barriers utilizing conventional health care. Many do not achieve their goals of normal blood sugar and blood pressure and remain at high risk for complications when compared with Whites. The discovery of newer approaches to improve their blood sugar and blood pressure control is needed. This study includes HCs, pharmacists and mobile health technology that

combined may make these support services more accessible to this vulnerable population. In addition, this study will examine the cost and cost-effectiveness of mHealth diabetes adherence support. The risks taken on behalf of subjects are small compared with the importance of the knowledge gained in this study.

Additional Benefits of mDAS Sleep Sub-Study Supplement:

Patients will have no direct benefit from participation in the sleep sub-study. However, it will contribute to scientific knowledge related to the challenges underserved African-American and Latino populations face in diabetes care, poor sleep duration and quality, which may contribute to worse diabetes-related health outcomes. Data exploring sleep quality within minority populations is very limited. Existing data suggest that African-Americans and Latinos are more likely to have poor sleep quality due to either psychosocial stress or non-standard sleep hours contributing to inconsistent sleep patterns and an increased risk of diabetes. Further sleep research involving minority populations with diabetes is needed for discovery of better treatment options.

At the end of the study, each participant will receive a thank you letter with a synopsis of their sleep results. Each participant will receive their average number of hours of sleep per night, the target number of hours of sleep they should aim for per night, and some general sleep hygiene tips.

8.0 Data Collection and Management Procedures

Physiological and Survey Measures: The UIC Center for Clinical Translational Science (CCTS) Clinical Interface Core (CIC) will provide data collection services for all physiologic measures. Trained study RAs will conduct all surveys by interview in the CIC, in English or Spanish. These staff will receive training and practice on specific techniques for establishing professional rapport, answering potential questions, and maintaining cooperation of respondents. Laminated cards with Likert-type scale responses are provided as a visual reference to patients. RAs possess laptop computers and remotely access REDCap for data entry. The survey requires an average duration of 60 minutes. Subjects will receive compensation as previously described (Section 6.2) for travel and time at each collection point. Public transit cards will be provided when needed.

Control Variables (Time-Constant/Baseline):

1. *Socio-demographics* will include age, gender, self-reported race and ethnicity, country of origin, income, highest level of education, employment status, global health status, and insurance.
2. *Diabetes and Medical History* will include time since diabetes diagnosis, receipt of diabetes education, current therapy, diabetes complications, and co-morbid conditions.

3. *Health Literacy* will be assessed using three screening questions with high discriminatory power among English and Spanish speaking populations.
4. *Mobile phone use* and comfort sending text messages will also be assessed using five items.
5. *Environmental Surveys* that assess loneliness, social cohesion, and neighborhood safety will be administered.

Additional Control Variable for mDAS sub-study

6. *Medication History* will include reported pain medications, sleep aids, or psychotropic medications prescribed and/or taken.

Intermediate Variables (Time-Varying): Intermediate variables will be collected at five time points (0, 6-, 12-, 18-, and 24-months).

1. *Perceived severity* of diabetes and *perceived susceptibility* will be assessed through two items adapted from Bradley.
2. *Perceived benefits of therapy* will be measured by the five-item survey related to benefits of therapy.
3. *Depression* will be measured using the nine-item Patient Health Questionnaire (PHQ-9).
4. *Social Support* will be measured using an assessment of amount of total support received and satisfaction of support from family, friends and healthcare team.
5. *Self-efficacy* will be measured by the Stanford 8-item self-efficacy for diabetes survey.

Process Measures:

1. *HC Activity* will be collected on a standardized worksheet completed after every participant contact (by phone and in-person). Information will include mode, time, and content of contact, results of glucose or blood pressure self-monitoring, goals, life events, pharmacist interactions, and interventions. Dr. Sharp will review HC worksheets for intervention fidelity.
2. *Text Messaging*: Detailed information on text messages sent and received are stored in a *mytapp* database. Drs. Gerber and Sharp will login to *mytapp* to review message data monthly to ensure fidelity. Additionally, Dr. Biggers will login to mytapp to review message data from the sub-study weekly.
3. *PCP and Pharmacist Activity*: The pharmacist will reconcile medications as part of the intervention, and the reconciled list from the medical chart will be used as data

to evaluate medication changes and overall complexity of therapy. Intensification of therapy will be defined as the number of increases in the dosage of antihypertensive, hypoglycemic agent, or insulin or the addition of a new agent since the baseline visit. Chart review will define the number of PCP visits and pharmacist videoconferences as well as the number of pharmacist- or physician-initiated medication changes.

4. *Patient Summary Evaluation*: Feedback will be collected from participants regarding their experience with the health coach, pharmacist, and videoconference technology. Individual responses will not be shared directly with the participant's health coach or pharmacist. Evaluation will be collected at 12 months for participants who receive the intervention in Year 1 or 24 months for those who receive it in Year 2.

Physiologic Outcomes and Quality of Life: Physiologic outcomes will be collected at five time points (0, 6-, 12-, 18-, and 24-months). CIC research staff will perform phlebotomy, blood pressure, weight and height recordings while being blinded to subject group assignment.

1. *Hemoglobin A1c* and *fasting lipid profile* (total cholesterol, HDL, LDL, and triglycerides) will be obtained via phlebotomy. The A1c laboratory test has National Glycohemoglobin Standardization Program certification.
2. A calibrated digital scale will measure *weight*.
3. At baseline, a height stadiometer will measure body *height*, with subjects removing their shoes (for BMI assessment).
4. *Blood pressure* will be assessed on subjects sitting down for at least five minutes, following standard procedure. Extreme blood pressures are reported to Dr. Gerber per CIC protocol.
5. *Health Related Quality of Life* will be measured using the EQ5-D (general) and DDS4 (diabetes specific).

Medication Adherence and Behavioral Outcomes: Medication adherence will be assessed using self-report and objective measures.

1. A self-report *medication adherence* survey will be included for all diabetes medications (developed by Lu et al, AIDS Behav 2008; Gonzalez JS et al, Diabetes Care, 2013). This instrument includes three items.
2. *Pharmacy fill data* will provide a more valuable method of adherence evaluation. Each participant will provide contact information for all pharmacies where prescriptions have been filled for the past two years. A signed HIPAA authorization form allows research personnel to contact pharmacies for medication fill information. For each prescription, the medication name, date dispensed, dosage,

frequency, quantity, and supply will be requested. The Proportion of Days Covered (PDC) will be calculated for all diabetes, hypertension, and hyperlipidemia medications.

3. The revised *Summary of Diabetes Self-Care Activities Measure (SDSCA)* will capture basic diet, exercise, blood sugar testing, foot care, and smoking with 11 core items.
4. *Alcohol misuse* will be assessed with the AUDIT-C brief screen. This instrument defines those with heavy/hazardous drinking behaviors.

Cost and Healthcare Utilization Data: We will conduct a cost-utility analysis comparing *mDAS* with usual care, following the guidelines for conducting pharmacoeconomic analyses by the Panel on Cost-Effectiveness and Medicine and the International Society for Pharmacoeconomics and Outcomes Research. We will conduct the analysis from the health-system perspective, and include the costs of running the program as well as the costs associated with patient health services utilization. Costs will include pharmacists and HCs (training and implementation), educational materials, tablet computers with data service, text messaging, and videoconferencing. Health services utilization will include costs associated with hospital admissions, emergency room visits, outpatient visits and prescription medications during the intervention period. Participants will be followed throughout the study to obtain data on their programmatic and healthcare services related costs. At data collection points, research assistants will obtain the necessary information from patients on healthcare use outside of UI Health. We will capture UI Health care information electronically from the hospital electronic records and billing data for enrolled subjects. We have previously analyzed similar UI Health data. Additional costs (salaries for HCs, etc.) will be estimated from prevailing internal costs at the time. Cost estimations for healthcare use will be based on national Medicare reimbursement rates (average DRG rates) for hospitalization, Medicare fee schedules for physician and other professional services, and Medicare's Resource-Based Relative Value Scale for outpatient procedures. Since our study will span a period exceeding one year, appropriate costs will be discounted and adjusted for inflation.

Data Management: We utilize REDCap (Vanderbilt University) for data entry. Our data manager is experienced in form creation, export, and randomization functions. Data will be exported, cleaned, and analyzed in SPSS, SAS, or R. To address missing data, we will examine the data for patterns of missingness and potential bias in missingness (SPSS Missing Values). If data are not missing at random, we will apply one of several available imputation methods in a sensitivity analysis based on the nature and extent of missing data. We will determine effectiveness using intention-to-treat principles with actual imputation of missing data. This will allow us to appreciate the potential biases inherent in a "real-world" setting due to drop out and poor adherence to study procedures.

9.0 Data Analysis

Dr. Yinglin Xia will lead analytic measures in collaboration with Dr. Michael Berbaum, IHRP Methodology Research Core. Cost effectiveness analyses will be conducted in collaboration with Dr. Kevin Stroupe, Loyola University. Sustainability and RE-AIM assessment will involve Dr. Paul Estabrooks, consultant. Drs. Gerber and Sharp (Co-PIs) will coordinate all analytic activities with regular meetings and keep data, statistical code, analytic output on a shared drive at IHRP. Additionally Dr. Biggers, along with the co-PIs will coordinate all analytic activities for the sub-study with regular meetings and keep data, statistical code, and analytic output on a shared drive at IHRP.

10.0 Quality Control and Quality Assurance

To continuously evaluate the fidelity of both conditions, the PIs will review written logs maintained by the pharmacists and HCs that include visit dates, length of visits, visit content, and disposition (e.g., visit completed, patient unreachable, etc.). Weekly group meetings with HCs and PIs provide an opportunity to monitor the intervention delivery and on-going training. All technology-related difficulties are reported to the coordinator immediately and discussed in weekly meetings with the PIs. Dr. Sharp will maintain weekly involvement with HCs to provide support and incorporate structured training opportunities quarterly. Additionally, Dr. Biggers will maintain weekly involvement with research personnel to provide support and incorporate structed training opportunities for the sub-study data collection and equipment retrieval.

11.0 Data and Safety Monitoring

The Data and Safety Monitoring Plan (DSMP) for the proposed project has two goals: (1) to insure the safety of the participants; and (2) to produce high quality research while considering both risks and benefits. Overall, the study poses minimal risk to participants. Because of this low risk status, the DSMP focuses on close monitoring by the co-principal investigators (co-PIs) and a safety officer (SO), along with prompt reporting of excessive adverse events and any serious adverse events to the NIH and to the IRB at the University of Illinois at Chicago (UIC).

Although there are additional reports to be produced by the study coordinator as a result of this DSMP, there are no substantive changes to the study protocol that might require review by the NIH. Safety reports will be sent to the study statistician, the co-PIs, and the SO. A research assistant will be responsible for assembling the data and producing these reports, as well as assuring that all parties obtain copies of these reports.

The research team will meet weekly and continuously evaluate the progress of the trial, including periodic assessments of data quality and timeliness, participant recruitment and retention, participant risk versus benefit, subject complaints (if any), adverse events, and other factors that can affect study outcomes. Summaries of enrollment and withdrawals will be reviewed together.

The frequency of data review differs according to the type of data as follows:

Data Type	Frequency of Review
Subject enrollment	Quarterly during recruitment phase
Subject status (retention)	Biannually
Adverse event rates (injuries)	As they occur

Qualifications and Responsibilities of the Safety Officer: We will identify an independent safety officer (SO) for this trial. This individual will have an advanced medical degree and experience in human subject protection, research study design, and the adverse event review process. The safety officer will review the reports sent by the study coordinator (at the frequency outlined above in the table) and will use a checklist to determine whether there is any corrective action, trigger, or an ad hoc review that should be communicated to the study investigator, the UIC IRB, and the NIH.

Reporting of Subject Accrual, Adherence to Inclusion/Exclusion Criteria: Review of the rate of subject accrual and adherence to inclusion/exclusion criteria will occur monthly during the recruitment phase by the co- PIs, SO, and the statistician. *Reporting of Adverse Events:* The research staff will be asked to report any problems the participants experience to the co-PIs either in person or by telephone. The safety officer (SO) will review, collate, and evaluate adverse events in real-time. Any adverse events reported to the IRB will also be reported to the NIH. All adverse events will be evaluated by the SO and the co-PIs within 72 hours; serious adverse events within 24 hours. Any study-related serious adverse event will be reported to the UIC IRB within 24 hours and the NIH within 2 weeks; all others will be included in the annual report to the NIH.

Data Privacy and Security: Formal training on data and participant safety is provided to all research staff involved in the data collection process, including health coaches. All staff and students involved in our research studies sign a confidentiality agreement stating that they are aware that any data they come in contact with is strictly confidential and is not to be discussed outside of the research project. They also complete the University of Illinois at Chicago online training on Human Subjects Protection (101) and the HIPAA Privacy Act. This will include the various modes of communication (e.g., telephone) and methods of maintaining confidentiality. At the UI Health and UIC research offices, all workstations require the use of a password in order to gain access. When leaving a workstation unattended, the user must log-out or lock their computer. Users are required to use screen savers that will be activated after ten minutes of inactivity and are password protected. All correspondence via fax must be stripped of patient identifiers. Before receiving a fax containing sensitive data, the sender must call the receiver to ensure the receiver is available to pick up the data. All e-mail with e-PHI will be sent securely through Cisco's Registered Envelope Service (CRES). The coding system will be kept in a locked cabinet or drawer separate from the actual data. Research investigators performing the statistical analyses data will not have access to information that can connect up the actual identity of a study participant and their data. Information connecting a study participant's name and study identifying number is kept in a locked file drawer in one of the co-PI's office.

VSee videoconferencing uses "peer-to-peer" sessions so information is not stored on a VSee server. VSee uses 256-bit AES (Advanced Encryption Standard) for secure transmission of information. Text messaging will not be secure and will not contain e-PHI. However, *mytapp* includes multiple security measures. Limited subject information will be available on *mytapp*, such as name and study ID number, and telephone number stored in an encrypted PostgreSQL database. We utilize TLS (Transport Layer Security) with encryption for all *mytapp* interactions and interactions with Twilio. All information from Twilio to *mytapp* is validated against embedded digital signatures to ensure authenticity and prevent "spoofing." A "PaperTrail" application provides a continuous audit trail to monitor *mytapp* use and all message activity. We will utilize a comprehensive approach to maximize privacy and data security for videoconferencing and text messaging services. We will include an information privacy and security committee to advise, assist, and monitor our research policies using technology and for documentation. The committee will include an IT security expert, privacy official, and a clinician from UI Health. The committee will: (1) conduct a formal risk analysis; (2) develop a risk management strategy based on risk analysis; (3) implement strategy and assist in training staff; and (4) monitor risk continuously.

12.0 Statistical Considerations

To address missing data, we will examine the data for patterns of missingness and potential bias in missingness (SPSS Missing Values). If data are not missing at random, we will apply one of several available imputation methods in a sensitivity analysis based on the nature and extent of missing data. We will determine effectiveness using intention-to-treat principles with actual imputation of missing data. This will allow us to appreciate the potential biases inherent in a "real-world" setting due to drop out and poor adherence to study procedures.

Patients who share a single PCP might exhibit similarities that are not shared with patients attended by other PCPs. To address this, we will include random effects in the model for clinic site, PCP, and HC (though the small numbers of HCs may call for a fixed effect approach, or "insufficient replication"). To examine a stricter examination of effect, we will also do a "per protocol" analysis of complete cases. Completed cases will not be dependent on intermediate time point data collections (6 and 18 months). Potential selection bias will be corrected by including in our models covariates that are differentially related to study participation.

Primary Outcomes

Univariate comparisons between the two study groups for outcomes and covariates at baseline will be conducted using chi-square tests for categorical variables, Kruskal-Wallis tests for ordinal variables, and t-tests for continuous variables. Non-normal continuous data will be transformed prior to analysis.

In order to provide comprehensive analysis for H_1, we will extend the usual analysis of crossover designs by including a longitudinal trend component in the first year. Thus, we can examine the time course (0, 6, and 12 months) as linear or quadratic over the first three

measurements. This will allow us to investigate whether changes are made early, and at what rate they continue through the rest of the time period. This analysis permits the comparison of trends between the two conditions. In addition, we will regard subjects as a random effect and will use Gaussian mixed-model estimation (SPSS MIXED). We can then substitute treatment by period interactions for the carry-over effects, and the model can be reduced in a recommended sequence (first omit carry-over, then omit treatment, finally omit period). SPSS MIXED will also be used to explore patterned covariance structures such as compound symmetry and autoregression along with incorporating time-constant and time-varying covariates, such as health literacy or diabetes distress.

The primary analysis of all physiological outcomes jointly in the repeated measures design will be conducted in a general linear model framework. SPSS MANOVA (for repeated measures) will be used to explore the simultaneous impact of the treatment on multiple correlated dependent variables, including the use of Roy-Bargmann stepdown F-tests and discriminant function analysis as post hoc tests to identify subsets of outcome measures affected. MANOVA secondary analyses will explore the impact on results of inhomogeneous baseline variables. Group by time-trend interaction contrasts will be used to explore different group trajectories of change. The potential consequences of medication intensification (e.g., initiation of insulin) will be explored by evaluating changes in BMI and quality of life. Exploratory subgroup analyses will follow a heterogeneity in treatment effects (HTE) framework. We will determine which subjects in the intervention group had the greatest improvement in outcomes, based on multiple pre-specified patient characteristics, including race (African-American or Latino), depression, comorbidities, baseline behaviors, health literacy, continuity of care, and social support. These analyses will also consider additional comparisons between subjects above and below median levels of videoconferencing and text messaging activity. Statistical tests will be implemented as interactions within the full data set.

If the intervention is effective, we will examine whether changes in self-efficacy, health beliefs, or social support serve as mediators for improved outcomes according to our conceptual framework. In addition, we will explore diabetes-related behaviors as well as medication treatment intensification as mediators using MPlus. To further identify the relative contributions of videoconferencing and/or text messaging, we will conduct meditational modeling with videoconferencing time, number of text messages, and HC contact time as mediating variables. This may describe any dose-response relationships between mHealth utilization and outcomes. We will compute bias-corrected bootstrap standard errors using MPlus. This offers accurate confidence intervals for mediation coefficients. Due to anticipated relationships between HC activity level and mHealth delivery, we plan to inspect correlations between mediators and incorporate any significant findings in model development. This analysis will demonstrate whether intervention effects are sensitive to mediators. Finally, given the sample size, observed (rather than latent) variables will be used in the mediation models. To enhance the power of mediation modeling, we will adopt an $\alpha=.10$ Type I error criterion to improve the chances of finding promising mediators for future study.

<u>Sustainability</u>

We expect a limited amount of decline in physiologic outcomes with the transition back to usual care. We will test the hypothesis that improvements in A1c, blood pressure, and LDL cholesterol will be maintained during the maintenance time period. The analyses will be conducted similarly as for H_1, and will evaluate change in the initial intervention group one year after intervention completion.

Consulting with Dr. Paul Estabrooks, we will implement a *Sustainability Action Plan (SAP)* to integrate HCs and ensure provision of pharmacy telehealth at an organizational level beyond the period of grant funding. The first phase will identify existing organizational goals and activities that support integration of HCs and pharmacy telehealth approaches into routine clinical operations. Our healthcare system is actively testing HC/peer educator models in multiple locations of the network. In collaboration with these sites and other key stakeholders, we will adapt an existing sustainability checklist to evaluate the presence of specific factors that are needed to support on-going operations within the organization. The next phase involves identifying existing and needed resources within our organization to support delivery of the additional services at the end of the study. The cost-effectiveness data (Aim 3) will help guide this procedure. Working with the Illinois Healthcare Reform Implementation Council and our existing relationship with the Illinois Community Health Worker Advisory Board (see letter of support), we will share details on training and reimbursement for community health workers (aka health coaches) as set forth in the Affordable Care Act and Illinois Bill HB5412 (the board will propose training and certification processes for community health workers and advice legislators). Dr. Edwin Fisher (consultant), as the Global Director of Peers for Progress, is actively involved in legislation to provide peer support service reimbursement and will aid in identifying relevant stakeholders. The final step focuses on executing the outlined action plan.

Table 4: RE-AIM Framework for Process Evaluation

**Reach**	Identify proportion of eligible population contacted, responded, participated, and excluded. Compare demographics and glycemic levels of eligible UI Health patients and study participants. Are the study participants representative of the clinical population?
**Effectiveness**	Determine change in hemoglobin A1c, blood pressure, and other outcomes (Aim 1). Are there unintended consequences of the intervention (e.g., negative impact on quality of life)?
**Adoption**	Conduct semi-structured interviews with randomly selected providers, staff, administrators, and health coaches across participating clinical sites (interview guides to be developed). Assess satisfaction and compare resources, setting, and intervention delivery across sites.
**Implementation**	Measure HC activity including encounters and phone calls completed, videoconferencing, and text messages delivered. Determine subject participation in intervention.
**Maintenance**	Implement Sustainability Action Plan (Aim 2). Evaluate change in outcomes one year after completion of the intervention and determine if positive outcomes were maintained. Assess drop-out rates and adverse events experienced.

Process evaluation will provide support for the project's potential as an intervention in a real-world setting. We will evaluate translation using the RE-AIM framework to determine dissemination potential and generalizability (Table 4). To assess adoption, we will conduct semi-structured interviews of providers, staff, and administrators, providing different perspectives.

Chain sampling will help identify those most likely to be "information rich" and continue until saturation reached.

Pharmacist interviews: Up to 15 pharmacists who have participated in the mHealth for Diabetes Adherence Support study (IRB 2016-0380) will be individually interviewed to gather feedback on delivering the intervention and potential for adoption.

An email invitation will be sent to pharmacists describing the purpose of the interview and a summary of the procedures. Interested pharmacists will be scheduled for one-on-one interviews with Dr. Sharp or a research assistant. The interviews will take place in private room at the pharmacist's clinical site or at a private campus location that is convenient for the pharmacist. A semi-structured interview guide will be used and content will focus on video conferencing, provider-pharmacist interactions, and interactions with health coaches. Interviews will be audio recorded. Names of the pharmacists will not be linked to their responses in order to maintain confidentiality. A brief demographic survey will be completed by the pharmacist and a number will be assigned to their interview. The interview will last approximately 30 minutes.

Interviews will be transcribed, coded and analyzed. Results will be used to inform the process evaluation component for the study (Aim 4) and future research involving clinical pharmacists, health coaches and the use of telehealth technology in health care.

<u>Cost and Cost Effectiveness</u>

We will obtain program and healthcare use data to estimate the cumulative costs from a health system perspective for each group over 24 months. To accommodate drop-outs and missing data (censoring) in the cost estimates, we will use a modification of survival analysis. To estimate average cumulative costs, we will divide the time interval following randomization into months, and then estimate cumulative costs to time T (i.e., 6, 12, 18, and 24 months) as: $C_{(T)} = p_t \times c_t$, where p_t is the probability of participating at month t, and c_t is the mean cost at month t given participation at t. Patients participating at the end of follow-up contribute cost information from randomization to end of follow-up, while patients who drop out contribute costs from randomization through drop-out date (and then zero costs thereafter until end of subject follow-up). We will then discount to estimate the average present value of costs: $C_{(T)} = \sum_{t=1}^{T} \hat{S}_t \overline{C}(t)/(1+r)^{t-1}$, where r is the discount rate and $\hat{S}_t$ is the Kaplan-Meier estimator of the probability of participation to month t. We will use a discount rate of 3% per year and calculate $C_{(T)}$ for subjects in both groups. For the cost-effectiveness analysis, we will calculate the incremental average costs resulting from *mDAS* vs. usual care and compare via a two-sample t-test for independent groups. Costs may not be normally distributed; however, if the sample is sufficiently large, t-tests on untransformed costs are appropriate.

Our effectiveness measures will include improvements in A1c, blood pressure, of cholesterol levels and projected quality-adjusted life-years (QALYs). Furthermore, a reduction in unnecessary healthcare utilization (emergency room visits and preventable hospitalizations) may result in cost savings. The incremental cost-effectiveness ratio (e.g., cost per change in A1c) of *mDAS* vs. usual care will be estimated at the end of 12 and 24 months. Cost per QALY will be determined from projections of continued mHealth involvement and assumptions concerning

the continued effects of *mDAS* on outcomes over time. The QALY and utility estimates will be based on the Markov model developed by the Diabetes Cost-effectiveness Group at the CDC (using UKPDS data). Sensitivity analyses will estimate the impact of changes in factors such as age, induced health-care visits, incidence of complications, HC costs, physician time, and discount rates. We will discount both QALYs and costs at 3% per year to maintain equivalent treatment of the measures of costs and effectiveness over time. To compare discounted QALYs, we will integrate the area under the Kaplan-Meier curves for each study condition to obtain mean QALYs. The difference between the two areas will provide the incremental effectiveness from receiving *mDAS*.

The ratio of net costs (Δ costs) and net utility (Δ utility) will yield the incremental cost utility ratio between the *mDAS* and usual care groups. One-way sensitivity analyses will determine the impact of discount rate (0%-10%) on the cost utility ratio. We will also examine variability associated with the cost-utility ratio using a bootstrapping technique. The bootstrapping results will be presented using the acceptability curve approach. The acceptability curve shows the proportion of samples in which the intervention is considered cost-effective relative to a comparison condition given a maximum amount that a decision maker is willing to pay for the outcome (e.g., $50,000/QALY). Specifically, Net Benefit = (-1) * (Net Costs) + (Net Effects) * (Willingness to pay) is calculated and the proportion of samples with mean net benefit greater than zero is plotted across a range of willingness to pay values. If *mDAS* lowers costs and produces more health it will tend to have an acceptability curve over 50% across all levels of willingness to pay.

<u>Sample Size Justification:</u> Sample size calculations are powered to detect change in A1c. The ADA Technical Review of Diabetes Self-Management Education reported that successful education programs achieved reductions of 5-20% in A1c.[113] The UKPDS 35 demonstrated that each 1% absolute reduction in mean A1c is associated with a 21% decrease in risk for any diabetes-related endpoint, a 21% decrease in diabetes-related deaths, a 14% decrease in risk for myocardial infarctions, and a 37% decrease in risk for microvascular complications. Based on our previous studies, we estimated a mean baseline A1c level of 10% with standard deviation of 1.8 and an effect size of (1.0/1.8) 0.56 for Aim 1. The cross-time correlation was estimated to be 0.30 with compound symmetry structure. We adjusted for clustering and assumed an intraclass correlation coefficient (ICC) of 0.01 with five clusters. This yielded a design effect of 1.34. Two-sided alpha of 0.05 and 80% power were assumed. Allowing for 20% drop-out, 220 patients will be required total.

UI Health billing data show there are 7,258 adult African-Americans and Latinos with diabetes. We estimate that approximately half of these patients have an A1c level above 8.0% based on prior study. We anticipate recruiting 6% of the eligible diabetic population. We have successfully recruited a similar size of patients with type 2 diabetes using comparable recruitment methods.

12.1 Additional Statistical Considerations for mDAS Sub-study

Dr. Biggers will receive statistical/analytic support from the Methodology Research Core at the Institute for Health Research and Policy (IHRP) and statistician Dr. Yinglin Xia. Descriptive statistics will be summarized as mean (standard deviation) or median (range) for continuous variables, and as frequencies (proportions) for categorical variables. We will address the three specific aims as follows:

Aim 1: Descriptive statistics will be calculated for the PROMIS as well as sleep duration based on text-message responses. Response rates will be quantified per individual and overall in aggregate. Interviews will provide an inventory of potential barriers to sleep in the home (e.g., presence of television in bedroom). These will be categorized and quantified. Differences in race/ethnicity within the descriptive data will also be compared.

Aim 2: Feasibility and acceptability of using the Actiwatch to collect objective sleep data will be based on the average number of days and amount of time per day the Actiwatch was worn. In addition, research personnel will obtain qualitative information regarding barriers to wearing the device in the semi-structured phone interview. Drs. Biggers, Sharp, and Gerber will collaboratively determine a coding scheme to describe issues discovered.

Aim 3: Independent and dependent variables will be explored via histograms to identify extreme values and assess normalcy (some Actiwatch data may yield spurious values). We will determine correlations between subjective text-message sleep diary and objective Actiwatch data (e.g., total sleep time and bouts of wakening after sleep onset). In addition, we will examine correlations between sleep parameters (e.g., total sleep time) and hemoglobin A1c, depression, quality of life, and sedentary behavior. For continuous variables, Pearson corrections will be calculated (and Spearman rank for non-parametric variables). Partial correlation models will include covariates such as patient age, gender, race/ethnicity, income, and education level. We will explore additional models that include the number of days the Actiwatch device was worn. All hypothesis tests will be 2-sided with a significance level of 0.05.

13.0 Regulatory Requirements

Informed Consent: Research assistants will obtain informed consent for all participants. They receive training that includes observation of the entire consent process prior to patient encounters. The informed consent document will be stored in a locked cabinet within IHRP, accessible to research assistants, projects coordinator, and Co-PIs. Our personnel include a bilingual research assistant who will consent all Spanish-speaking participants.

Subject Confidentiality: All research staff, including pharmacists and HCs, will have formal IRB human subject protection and HIPAA training. Additional HC training includes discussions on maintaining confidentiality of health information, including communication with health providers. For data collection, all subjects will be assigned a blind code number. These lists will be kept in a locked file and will not be shown to staff. Data collected from computer use files will

have the name removed and the code number attached by the research coordinator. Project staff who have access to the data will not have access to subject names. No clinical staff who have direct contact with the subjects will have access to research data. Finally, there will be no personal information stored on the server itself; and all e-mail communication will be encrypted and secure. During data analysis, when data integrity has been confirmed, identifiers will be destroyed. PHI will be included to the extent needed for study objectives, including medication use, and patients will provide consent for release.

References

1. Stading JA, Phan L, Walter A, Bilslend L, White R, Qi Y. Initial experience of clinical pharmacy services delivered by computer communications via Cisco Jabber Video in a US Veterans Administration Medical Center. Journal of Pharmacy Technology 2013;30:76-80.
2. Newsome J. Virtual medication therapy management: the cornerstone to a pharmacy renaissance. J Am Pharm Assoc (2003) 2013;53:159-62.
3. Hamine S, Gerth-Guyette E, Faulx D, Green BB, Ginsburg AS. Impact of mHealth chronic disease management on treatment adherence and patient outcomes: a systematic review. J Med Internet Res 2015;17:e52.
4. Bluml BM, Watson LL, Skelton JB, Manolakis PG, Brock KA. Improving outcomes for diverse populations disproportionately affected by diabetes: final results of Project IMPACT: Diabetes. J Am Pharm Assoc (2003) 2014;54:477-85.
5. Little R, Rubin D. Statistical Analysis with Missing Data. Hoboken, NJ: John Wiley & Sons Inc; 2014.
6. Cole-Lewis H, Kershaw T. Text messaging as a tool for behavior change in disease prevention and management. Epidemiologic Reviews 2010;32:56-69.
7. Spark LC, Fjeldsoe BS, Eakin EG, Reeves MM. Efficacy of a text message-delivered extended contact intervention on maintenance of weight loss, physical activity, and dietary behavior change. JMIR mHealth and UHealth 2015;3:e88; 1-15.
8. Stark Casagrande S, Fradkin JE, Saydah SH, Rust KF, Cowie CC. The prevalence of meeting A1C, blood pressure, and LDL goals among people with diabetes, 1988–2010. Diabetes Care 2013.
9. Centers for Disease Control and Prevention. National diabetes statistics report: estimates of diabetes and its burden in the United States, 2014. Atlanda, GA: Centers for Disease Control and Prevention; 2014.
10. Home P. The challenge of poorly controlled diabetes mellitus. Diabetes & Metabolism 2003;29:101-9.
11. Cowie CC, Rust KF, Byrd-Holt DD, et al. Prevalence of diabetes and impaired fasting glucose in adults in the U.S. population: National Health And Nutrition Examination Survey 1999-2002. Diabetes Care 2006;29:1263-8.
12. Harris MI. Racial and ethnic differences in health care access and health outcomes for adults with type 2 diabetes. Diabetes Care 2001;24:454-9.
13. Braun R, Catalani C, Wimbush J, Israelski D. Community health workers and mobile technology: a systematic review of the literature. PLoS One 2013;8:e65772.
14. Fortney JC, Burgess JF, Jr., Bosworth HB, Booth BM, Kaboli PJ. A re-conceptualization of access for 21st century healthcare. J Gen Intern Med 2011;26 Suppl 2:639-47.
15. Demiris G, Afrin LB, Speedie S, et al. Patient-centered applications: use of information technology to promote disease management and wellness. A white paper by the AMIA knowledge in motion working group. J Am Med Inform Assoc 2008;15:8-13.

16. Gerber BS, Cano AI, Caceres ML, et al. A pharmacist and health promoter team to improve medication adherence among Latinos with diabetes. The Annals of Pharmacotherapy 2010;44:70-9.
17. Minino AM. Death in the United States, 2009. NCHS data brief 2011;(64):1-8.
18. Heisler M, Smith DM, Hayward RA, Krein SL, Kerr EA. Racial disparities in diabetes care processes, outcomes, and treatment intensity. Medical Care 2003;41:1221-32.
19. Heisler M, Faul JD, Hayward RA, Langa KM, Blaum C, Weir D. Mechanisms for racial and ethnic disparities in glycemic control in middle-aged and older Americans in the health and retirement study. Arch Intern Med 2007;167:1853-60.
20. Kirk JK, Bell RA, Bertoni AG, et al. Ethnic disparities: control of glycemia, blood pressure, and LDL cholesterol among US adults with type 2 diabetes. The Annals of Pharmacotherapy 2005;39:1489-501.
21. Kirk JK, D'Agostino RB, Jr., Bell RA, et al. Disparities in HbA1c levels between African-American and non-Hispanic white adults with diabetes: a meta-analysis. Diabetes Care 2006;29:2130-6.
22. Kirk JK, Passmore LV, Bell RA, et al. Disparities in A1C levels between Hispanic and non-Hispanic white adults with diabetes: a meta-analysis. Diabetes Care 2008;31:240-6.
23. Carter JS, Pugh JA, Monterrosa A. Non-insulin-dependent diabetes mellitus in minorities in the United States. Annals of Internal Medicine 1996;125:221-32.
24. Perneger TV, Brancati FL, Whelton PK, Klag MJ. End-stage renal disease attributable to diabetes mellitus. Annals of Internal Medicine 1994;121:912-8.
25. Garfield SA, Malozowski S, Chin MH, et al. Considerations for diabetes translational research in real-world settings. Diabetes Care 2003;26:2670-4.
26. American Diabetes Association. Standards of Medical Care in Diabetes—2013. Diabetes Care 2013;36:S11-S66.
27. Adherence to Long-Term Therapies; Evidence for Action. Geneva, Switzerland: World Health Organization; 2003.
28. Nelson KM, Reiber G, Boyko EJ. Diet and exercise among adults with type 2 diabetes: findings from the third national health and nutrition examination survey (NHANES III). Diabetes Care 2002;25:1722-8.
29. Osterberg L, Blaschke T. Adherence to medication. N Engl J Med 2005;353:487-97.
30. Haynes RB, Ackloo E, Sahota N, McDonald HP, Yao X. Interventions for enhancing medication adherence. Cochrane Database Syst Rev 2008:CD000011.
31. Viswanathan M, Golin CE, Jones CD, et al. Interventions to improve adherence to self-administered medications for chronic diseases in the United States: a systematic review. Ann Intern Med 2012;157:785-95.
32. Fischer MA, Stedman MR, Lii J, et al. Primary medication non-adherence: analysis of 195,930 electronic prescriptions. J Gen Intern Med 2010;25:284-90.
33. Wadden TA, Stunkard AJ. Handbook of obesity treatment. New York: Guilford Press; 2002.
34. Huang ES, Brown SE, Thakur N, et al. Racial/ethnic differences in concerns about current and future medications among patients with type 2 diabetes. Diabetes Care 2009;32:311-6.

35. Gellad WF, Haas JS, Safran DG. Race/ethnicity and nonadherence to prescription medications among seniors: results of a national study. J Gen Intern Med 2007;22:1572-8.
36. Monane M, Bohn RL, Gurwitz JH, Glynn RJ, Levin R, Avorn J. Compliance with antihypertensive therapy among elderly Medicaid enrollees: the roles of age, gender, and race. Am J Public Health 1996;86:1805-8.
37. Bosworth HB, Powers B, Grubber JM, et al. Racial differences in blood pressure control: potential explanatory factors. J Gen Intern Med 2008;23:692-8.
38. Clark LT, Maki KC, Galant R, Maron DJ, Pearson TA, Davidson MH. Ethnic differences in achievement of cholesterol treatment goals. Results from the National Cholesterol Education Program Evaluation Project Utilizing Novel E-Technology II. J Gen Intern Med 2006;21:320-6.
39. Kaplan RC, Bhalodkar NC, J BE, Jr., White J, Brown DL. Race, ethnicity, and sociocultural characteristics predict noncompliance with lipid-lowering medications. Prev Med 2004;39:1249-55.
40. Kripalani S, Yao X, Haynes RB. Interventions to enhance medication adherence in chronic medical conditions: a systematic review. Arch Intern Med 2007;167:540-50.
41. Bogner HR, de Vries HF. Integrating type 2 diabetes mellitus and depression treatment among African Americans: a randomized controlled pilot trial. Diabetes Educ 2010;36:284-92.
42. Patient-centered Primary Care Collaborative. 2013. Available at http://www.pcpcc.org/.
43. Rittenhouse DR, Shortell SM. The patient-centered medical home: will it stand the test of health reform? JAMA 2009;301:2038-40.
44. Iyer R, Coderre P, McKelvey T, et al. An employer-based, pharmacist intervention model for patients with type 2 diabetes. American Journal of Health-System Pharmacy: Official Journal of the American Society of Health-System Pharmacists 2010;67:312-6.
45. Rochester CD, Leon N, Dombrowski R, Haines ST. Collaborative drug therapy management for initiating and adjusting insulin therapy in patients with type 2 diabetes mellitus. American Journal of Health-System Pharmacy: Official Journal of the American Society of Health-System Pharmacists 2010;67:42-8.
46. Taveira TH, Friedmann PD, Cohen LB, et al. Pharmacist-led group medical appointment model in type 2 diabetes. The Diabetes Educator 2010;36:109-17.
47. Nieuwlaat R, Wilczynski N, Navarro T, et al. Interventions for enhancing medication adherence. Cochrane Database Syst Rev 2014;11:CD000011.
48. Viswanathan M, Kahwati LC, Golin CE, et al. Medication therapy management interventions in outpatient settings: a systematic review and meta-analysis. JAMA Internal Medicine 2015;175:76-87.
49. Chung N, Rascati K, Lopez D, Jokerst J, Garza A. Impact of a clinical pharmacy program on changes in hemoglobin A1c, diabetes-related hospitalizations, and diabetes-related emergency department visits for patients with diabetes in an underserved population. Journal of Managed Care and Speciality Pharmacy 2014;20:914-9.
50. National Center for Chronic Disease Prevention and Health Promotion. Select Features of State Pharmacist Collaborative Practice Laws. Atlanta, GA: Center For Disease Control; 2013.

51. Walsh JM, McDonald KM, Shojania KG, et al. Quality improvement strategies for hypertension management: a systematic review. Med Care 2006;44:646-57.
52. Lee SY, Bender DE, Ruiz RE, Cho YI. Development of an easy-to-use Spanish Health Literacy test. Health Serv Res 2006;41:1392-412.
53. Community Preventative Services Task Force. Team-based care to improve blood pressure control. Recommendation of the Community Preventative Services Task Force. American Journal of Preventative Medicine 2014;47(1):100-2.
54. Margolis KL, Asche SE, Bergdall AR, et al. Effect of home blood pressure telemonitoring and pharmacist management on blood pressure control: a cluster randomized clinical trial. JAMA 2013;310:46-56.
55. Antoine S-L, Pieper D, Mathes T, Eikermann M. Improving the adherence of type 2 diabetes mellitus patients with pharmacy care: a systematic review of randomized controlled trials. BMC Endocrine Disorders 2014;14:1-8.
56. Gary TL, Batts-Turner M, Yeh HC, et al. The effects of a nurse case manager and a community health worker team on diabetic control, emergency department visits, and hospitalizations among urban African Americans with type 2 diabetes mellitus: a randomized controlled trial. Archives of Internal Medicine 2009;169:1788-94.
57. Babamoto KS, Sey KA, Camilleri AJ, Karlan VJ, Catalasan J, Morisky DE. Improving diabetes care and health measures among hispanics using community health workers: results from a randomized controlled trial. Health Educ Behav 2009;36:113-26.
58. Lujan J, Ostwald SK, Ortiz M. Promotora diabetes intervention for Mexican Americans. Diabetes Educator 2007;33:660-70.
59. Ingram M, Gallegos G, Elenes J. Diabetes is a community issue: the critical elements of a successful outreach and education model on the U.S.-Mexico border. Preventing Chronic Disease 2005;2:A15.
60. Corkery E, Palmer C, Foley ME, Schechter CB, Frisher L, Roman SH. Effect of a bicultural community health worker on completion of diabetes education in a Hispanic population. Diabetes Care 1997;20:254-7.
61. DePue JD, Dunsiger S, Seiden AD, et al. Nurse-community health worker team improves diabetes care in American Samoa: results of a randomized controlled trial. Diabetes Care 2013;36:1947-53.
62. Rothschild SK, Martin MA, Swider SM, et al. Mexican American trial of community health workers: a randomized controlled trial of a community health worker intervention for Mexican Americans with type 2 diabetes mellitus. Am J Public Health 2013.
63. Thom DH, Ghorob A, Hessler D, De Vore D, Chen E, Bodenheimer TA. Impact of peer health coaching on glycemic control in low-income patients with diabetes: a randomized controlled trial. Ann Fam Med 2013;11:137-44.
64. Peers for Progress. Opportunities for Peer Support in the Affordable Care Act. Leawood, KS: National Counsil of La Raza; 2012 December 2012.
65. Free C, Phillips G, Watson L, et al. The effectiveness of mobile-health technologies to improve health care service delivery processes: a systematic review and meta-analysis. PLoS Medicine 2013;10:e1001363.

66. Gentles SJ, Lokker C, McKibbon KA. Health information technology to facilitate communication involving health care providers, caregivers, and pediatric patients: a scoping review. J Med Internet Res 2010;12:e22.
67. Sutcliffe P, Martin S, Sturt J, et al. Systematic review of communication technologies to promote access and engagement of young people with diabetes into healthcare. BMC Endocrine Disorders 2011;11:1.
68. Worswick J, Wayne SC, Bennett R, et al. Improving quality of care for persons with diabetes: an overview of systematic reviews - what does the evidence tell us? Systematic Reviews 2013;2:26.
69. Zhai YK, Zhu WJ, Cai YL, Sun DX, Zhao J. Clinical and cost-effectiveness of telemedicine in type 2 diabetes mellitus: a systematic review and meta-analysis. Medicine 2014;93:e312.
70. Verhoeven F, van Gemert-Pijnen L, Dijkstra K, Nijland N, Seydel E, Steehouder M. The contribution of teleconsultation and videoconferencing to diabetes care: a systematic literature review. J Med Internet Res 2007;9:e37.
71. Piette JD. Interactive behavior change technology to support diabetes self-management: where do we stand? Diabetes Care 2007;30:2425-32.
72. Eysenbach G. The law of attrition. J Med Internet Res 2005;7:e11.
73. Krishna S, Boren SA. Diabetes self-management care via cell phone: a systematic review. Journal of Diabetes Science and Technology 2008;2:509-17.
74. Liang X, Wang Q, Yang X, et al. Effect of mobile phone intervention for diabetes on glycaemic control: a meta-analysis. Diabetic Medicine 2011;28:455-63.
75. Dick JJ, Nundy S, Solomon MC, Bishop KN, Chin MH, Peek ME. Feasibility and usability of a text message-based program for diabetes self-management in an urban African-American population. Journal of Diabetes Science and Technology 2011;5:1246-54.
76. Franklin B, Plum T. Successful web survey methodologies for measuring the impact of networked electronic services (MINES for Libraries). IFLA Journal 2006;32:28-40.
77. Herbert L, Owen V, Pascarella L, Streisand R. Text message interventions for children and adolescents with type 1 diabetes: a systematic review. Diabetes Technol Ther 2013;15:362-70.
78. de Jongh T, Gurol-Urganci I, Vodopivec-Jamsek V, Car J, Atun R. Mobile phone messaging for facilitating self-management of long-term illnesses. Cochrane Database Syst Rev 2012;12:CD007459.
79. Kannisto KA, Koivunen MH, Välimäki MA. Use of mobile phone text message reminders in health care services: a narrative literature review. Journal of Medical Internet Research 2014;16:e222.
80. Chow CK, Redfern J, Hillis GS, et al. Effect of lifestyle-focused text messaging on risk factor modification in patients with coronary heart disease: A randomized clinical trial. JAMA 2015;314:1255-63.
81. Tomlinson M, Rotheram-Borus MJ, Swartz L, Tsai AC. Scaling up mHealth: where is the evidence? PLoS Medicine 2013;10:e1001382.
82. Head KJ, Noar SM, Iannarino NT, Grant Harrington N. Efficacy of text messaging-based interventions for health promotion: A meta-analysis. Social Science & Medicine 2013;97:41-8.

83. Kallander K, Tibenderana JK, Akpogheneta OJ, et al. Mobile health (mHealth) approaches and lessons for increased performance and retention of community health workers in low- and middle-income countries: a review. J Med Internet Res 2013;15:e17.
84. Heisler M, Choi H, Palmisano G, et al. Comparison of community health worker-led diabetes medication decision-making support for low-income Latino and African American adults with diabetes using e-health tools versus print materials: a randomized, controlled trial. Ann Intern Med 2014;161:S13-22.
85. Shaw R, Bosworth H. Short message service (SMS) text messaging as an intervention medium for weight loss: A literature review. Health Informatics Journal 2012;18:235-50.
86. Stephens J, Allen J. Mobile phone interventions to increase physical activity and reduce weight: a systematic review. J Cardiovasc Nurs 2013;28:320-9.
87. Holtz B, Lauckner C. Diabetes management via mobile phones: a systematic review. Telemed J E Health 2012;18:175-84.
88. Siriwardena LS, Wickramasinghe WA, Perera KL, Marasinghe RB, Katulanda P, Hewapathirana R. A review of telemedicine interventions in diabetes care. J Telemed Telecare 2012;18:164-8.
89. Gerber BS, Rapacki L, Castillo A, et al. Design of a trial to evaluate the impact of clinical pharmacists and community health promoters working with African-Americans and Latinos with diabetes. BMC Public Health 2012;12:891.
90. Rojas E, Gerber BS, Tilton J, Rapacki L, Sharp LK. Pharmacists' perspectives on collaborating with community health workers in diabetes care. J Am Pharm Assoc (2003) 2015;55:429-33.
91. Gerber B, Schiffer L, Brown AAB, M. L. Rimmer, J. H., Braunschweig CL, Fitzgibbon ML. Video telehealth for weight management of African-American women. J Telemed Telecare 2013;19:266-72.
92. Rimmer JH, Wang E, Pellegrini CA, Lullo C, Gerber BS. Telehealth weight management intervention for adults with physical disabilities: a randomized controlled trial. Am J Phys Med Rehabil 2013;92:1084-94.
93. Gerber BS, Stolley MR, Thompson AL, Sharp LK, Fitzgibbon ML. Mobile phone text messaging to promote healthy behaviors and weight loss maintenance: a feasibility study. Health Informatics Journal 2009;15:17-25.
94. Nundy S, Razi RR, Dick JJ, et al. A text messaging intervention to improve heart failure self-management after hospital discharge in a largely African-American population: before-after study. J Med Internet Res 2013;15:e53.
95. Rosenstock IM, Strecher VJ, Becker MH. Social learning theory and the Health Belief Model. Health Educ Q 1988;15:175-83.
96. Hill-Briggs F. Problem solving in diabetes self-management: a model of chronic illness self-management behavior. Ann Behav Med 2003;25:182-93.
97. Curioso WH, Alex Quistberg D, Cabello R, et al. "It's time for your life": How should we remind patients to take medicines using short text messages? AMIA Annual Symposium proceedings / AMIA Symposium AMIA Symposium 2009;2009:129-33.
98. Reach G. Patient non-adherence and healthcare-provider inertia are clinical myopia. Diabetes Metab 2008;34:382-5.

99. Binik A, Weijer C, McRae AD, et al. Does clinical equipoise apply to cluster randomized trials in health research? Trials 2011;12:118.
100. Pew Internet & American Life Project Commentary: Internet and cell phone facts. Available at: http://www.pewinternet.org/fact-sheets/health-fact-sheet/
101. Pew Internet & American Life Project Commentary: Smartphone ownership, 2013. Available at: *http://www.pewinternet.org/reports/smartphone-ownership-2013.aspx*

Additional References for *mDAS* Sleep Sub-Study

1. Gozashti, Eslami, N., Radfar, M. H. & Pakmanesh, H. Sleep Pattern, Duration and Quality in Relation with Glycemic Control in People with Type 2 Diabetes Mellitus. *Iranian Journal of Medical Sciences* **41,** 531–538 (2016).
2. Knutson, K., Ryden, A., Mander, B. & Cauter, E. Role of Sleep Duration and Quality in the Risk and Severity of Type 2 Diabetes Mellitus. *Arch Intern Med* **166,** 1768–1774 (2006).
3. Knutson, K., Cauter, E., Zee, P., Liu, K. & Lauderdale, D. Cross-Sectional Associations Between Measures of Sleep and Markers of Glucose Metabolism Among Subjects With and Without Diabetes. *Diabetes Care* **34,** 1171–1176 (2011).
4. Chen, X. *et al.* Racial/Ethnic Differences in Sleep Disturbances: The Multi-Ethnic Study of Atherosclerosis (MESA). *Sleep* (2015). doi:10.5665/sleep.4732
5. Papp, K. K., Penrod, C. E. & Strohl, K. P. Knowledge and attitudes of primary care physicians toward sleep and sleep disorders. *Sleep Breath* **6,** 103–9 (2002).
6. Unknown. Statistics About Diabetes_ American Diabetes Association®.pdf.
7. Spanakis, E. & Golden, S. Race/Ethnic Difference in Diabetes and Diabetic Complications. *Curr Diabetes Rep* **13,** 814–23 (2013).
8. Group, D. *et al.* Factors Affecting the Decline in Incidence of Diabetes in the Diabetes Prevention Program Outcomes Study (DPPOS). *Nestle Nutr Works Se* **64,** 989–998 (2015).
9. Spiegel, K., Leproult, R. & Cauter, E. Impact of sleep debt on metabolic and endocrine function. *Lancet* **354,** 1435–1439 (1999).
10. Cauter, E. *et al.* Impact of Sleep and Sleep Loss on Neuroendocrine and Metabolic Function. *Hormone Res Paediatr* **67,** 2–9 (2007).
11. Ohkuma, T. *et al.* U-shaped association of sleep duration with metabolic syndrome and insulin resistance in patients with type 2 diabetes: The Fukuoka Diabetes Registry. *Metabolis* **63,** 484–491 (2014).
12. Societies, L. *et al.* The Contribution of Psychosocial Stressors to Sleep among African Americans in the Jackson Heart Study. *Sleep* **39,** 1411–9 (2016).
13. Bakker, J. *et al.* Associations between Obstructive Sleep Apnea, Sleep Duration, and Abnormal Fasting Glucose. The Multi-Ethnic Study of Atherosclerosis. *Am J Resp Crit Care* **192,** 745–753 (2015).
14. Presser, H. B. & Ward, B. W. Nonstandard work schedules over the life course: a first look. *Monthly Labor Review* (2011).
15. Morris, C. *et al.* Endogenous circadian system and circadian misalignment impact glucose tolerance via separate mechanisms in humans. *Proc National Acad Sci* **112,** E2225–E2234 (2015).

16. Gan, Y. *et al.* Shift work and diabetes mellitus: a meta-analysis of observational studies. *Occup Environ Med* **72,** oemed–2014–102150 (2014).
17. Morris, C., Purvis, T., Mistretta, J. & Scheer, F. Effects of the internal circadian system and circadian misalignment on glucose tolerance in chronic shift workers. *The Journal of Clinical Endocrinology & Metabolism* **101,** 1066–1074 (2016).
18. Vimalananda, V. *et al.* Night-shift work and incident diabetes among African-American women. *Diabetologia* **58,** 699–706 (2015).
19. Roy, T. & Lloyd, C. Epidemiology of depression and diabetes: A systematic review. *J Affect Disorders* **142,** S8–S21 (2012).
20. Ali, S. *et al.* The association between depression and health-related quality of life in people with type 2 diabetes: a systematic literature review. *Diabetes Metabolism Res Rev* **26,** 75–89 (2010).
21. Lakerveld, J. *et al.* The relation between sleep duration and sedentary behaviours in European adults. *Obes Rev* **17,** 62–67 (2016).
22. Healy, G. N. *et al.* Objectively Measured Sedentary Time, Physical Activity, and Metabolic Risk: The Australian Diabetes, Obesity and Lifestyle Study (AusDiab). *Diabetes Care* **31,** 369–371 (2008).
23. Luyster, F. & Dunbar-Jacob, J. Sleep Quality and Quality of Life in Adults With Type 2 Diabetes. *Diabetes Educ* **37,** 347–355 (2011).
24. Goldney, R., Phillips, P., Fisher, L. & Wilson, D. Diabetes, Depression, and Quality of Life A population study. *Diabetes Care* **27,** 1066–1070 (2004).
25. George, E., Rosenkranz, R. & Kolt, G. Chronic disease and sitting time in middle-aged Australian males: findings from the 45 and Up Study. *Int J Behav Nutr Phy* **10,** 1–8 (2013).
26. Hu, F. *et al.* Physical Activity and Television Watching in Relation to Risk for Type 2 Diabetes Mellitus in Men. *Arch Intern Med* **161,** 1542–1548 (2001).
27. Kushida, C. *et al.* Comparison of actigraphic, polysomnographic, and subjective assessment of sleep parameters in sleep-disordered patients. *Sleep Med* **2,** 389–396 (2001).
28. Mantua, J., Gravel, N. & Spencer, R. Reliability of Sleep Measures from Four Personal Health Monitoring Devices Compared to Research-Based Actigraphy and Polysomnography. *Sensors-basel* **16,** 646 (2016).
29. Zambotti, M., Claudatos, S., Inkelis, S., Colrain, I. & Baker, F. Evaluation of a consumer fitness-tracking device to assess sleep in adults. *Chronobiol Int* **32,** 1024–1028 (2015).
30. Martin, J. & Hakim, A. Wrist Actigraphy. *Chest* **139,** 1514–1527 (2011).
31. Nagappa, M. *et al.* Validation of the STOP-Bang Questionnaire as a Screening Tool for Obstructive Sleep Apnea among Different Populations: A Systematic Review and Meta-Analysis. *Plos One* **10,** e0143697 (2015).
32. Chiu, H.-Y. *et al.* Diagnostic Accuracy of the Berlin Questionnaire, STOP-Bang, STOP, and Epworth Sleepiness Scale in Detecting Obstructive Sleep Apnea: A Bivariate Meta-analysis. *Sleep Med Rev* (2016). doi:10.1016/j.smrv.2016.10.004
33. Ferri, R. *et al.* A single question for the rapid screening of restless legs syndrome in the neurological clinical practice. *Eur J Neurol* **14,** 1016–1021 (2007).

34. Walters, A. *et al.* Review of Diagnostic Instruments for the Restless Legs Syndrome/Willis-Ekbom Disease (RLS/WED): Critique and Recommendations. *J Clin Sleep Med* (2014). doi:10.5664/jcsm.4298
35. Carney, C. *et al.* The Consensus Sleep Diary: Standardizing Prospective Sleep Self-Monitoring. *Sleep* (2012). doi:10.5665/sleep.1642
36. Mollayeva, T. *et al.* The Pittsburgh sleep quality index as a screening tool for sleep dysfunction in clinical and non-clinical samples: A systematic review and meta-analysis. *Sleep Medicine Reviews* **25,** 52–73 (2016).
37. Polonsky, W. *et al.* Assessing psychosocial distress in diabetes: development of the diabetes distress scale. *Diabetes Care* **28,** 626–31 (2005).
38. Gusi, N., Olivares, P. R. & Rajendram, R. The EQ-5D Health-Related Quality of Life Questionnaire. *Handbook of Disease Burdens and Quality of Life Measures* 87–99 (2010). doi:10.1007/978-0-387-78665-0_5
39. Kroenke, K., Spitzer, R. L. & Williams, J. B. W. The PHQ-9: Validity of a Brief Depression Severity Measure. *Journal of General Internal Medicine* **16,** 606–613 (2001).
40. Toobert, D., Hampson, S. & Glasgow, R. The summary of diabetes self-care activities measure: results from 7 studies and a revised scale. *Diabetes Care* **23,** 943–950 (2000).
41. SAS® *Software. SAS and all other SAS Institute Inc. product or service names are registered trademarks or trademarks of SAS Institute Inc. in the USA and other countries. ® indicates USA registration.*

PHARMACY MANAGEMENT PROCOTOL

OBJECTIVES

A. Maximize the benefits of medication therapy management

B. Improve and sustain an acceptable patient adherence to their drug regimen

C. Provide education to patients' and caregivers about diseases states and medications

D. Provide consultative services to physicians and other healthcare providers regarding medication issues

E. Assist in coordination of care with other healthcare providers as it relates to the patient's drug therapy and overall health

F. Improve healthcare utilization and minimize the individual's healthcare cost

G. Identify, monitor for, and prevent or minimize adverse drug events and medication related complications

H. Prevent duplication of drug therapy, and avoidable drug interactions

I. Assist physicians in monitoring patients drug therapy and medical conditions, particularly their response to therapy in-between provider visits

J. Conduct research regarding medication therapy management and related issues

K. Serve as an education site for Pharm.D. students, PGY1 and PGY2 residents and fellows

PROCEDURES

A. Appointment Scheduling

1. New patients will be scheduled for an initial videoconference with pharmacist after health coach (HC) has completed at least one home visit with patient.
2. The HC will schedule the initial telehealth pharmacy visit within 90 days of a patient's enrollment in the study. Initial visits will be scheduled for 60 minutes. Scheduling will be done according to each recruitment site's preference.
    a. Medication Therapy Management Clinic (MTMC): Visits will be scheduled in the electronic Medication Management Therapy Clinic (MTMC) appointment book in Cerner.

For instructions on scheduling, please refer to the MTMC Scheduling Guide (Appendix A). Appointment times will be based on patient room availability as well as the patient's and HC's availability. Only one research telehealth patient visit may be scheduled per half day (between the hours of 9am-12pm and 1pm – 4 pm. The pharmacist assigned to the patient at their initial visit will continue to see them for the duration of their time in the study, when possible.

b. Mile Square: HC will forward a clinic note to the pharmacist with a message requesting an appointment. The HC will also text the pharmacist to alert them that a telehealth visit is needed. The pharmacist will work with the HC to find an agreeable time and will have that time blocked in the appointment book in Cerner.

c. Family Medicine (University Village) – HCs will schedule appointments directly with IRB approved pharmacists via encrypted email (preferred) or phone. Pharmacists will keep a separate calendar and block the time in their Cerner appointment book.

3. Subsequent visits will be scheduled by the pharmacist at each telehealth visit, based on the needs of the patient, as well as the availability of the patient and HC. These visits will be scheduled for a minimum of 30 minutes. However, visits with Spanish-speaking patients should be scheduled for 60 minutes to allow for translation.

4. Encounter cancellations will be made as soon as possible (*at least 24 hours prior to scheduled time)* when possible. The HC will call or text the patient the day before to confirm the visit. HCs may reschedule visits at the patient's request, but must work with the pharmacist to ensure their availability.

5. If the cancellation occurs on the same day as the scheduled telehealth visit, the HC will email the pharmacist(s) as soon as possible to free up the appointment for walk-in patients in the clinic. The health coach may also call or text the pharmacists if they are at a patient's home and email is not available to them. In some cases, patients may confirm that they are available the night before but not be present or willing to conduct the visit when the health coach presents the

following day. Nonetheless, pharmacists will be informed as soon as possible.

    a. In the case of MTMC and Family Medicine, calls to the main clinic line are preferred.

6. If a patient strongly prefers to see the pharmacist in-person instead of by videoconference they may do so and remain in the study. The HC will accompany the patient to the visit as done in the previous Diabetes Health protocol.
7. If the patient declines videoconferencing with a pharmacist after enrolling in the study, the HC will offer a pharmacy visit periodically during the remainder of the study. The patient may remain in the study and continue to receive the HC intervention only.
8. If a patient fails a scheduled clinic visit or cannot be reached (e.g., not home at scheduled appointment time) adequate follow-up will be ensured by taking the following steps:
    a. The HC will leave an enclosed “Sorry we missed you” note at the patient’s home with the HC’s number to call and reschedule.
    b. The HC will text or phone the patient the day before each scheduled visit to confirm and again the day of the visit. The HC is responsible for rescheduling the appointment. The HC may also contact the emergency contacts listed in the patient’s study contact form in efforts to reach the patient.
    c. The HC may also review the patient’s upcoming appointments at other UI Health departments, and try to meet the patient at one of their scheduled appointments to follow-up and reschedule the visit if possible.
    d. If the patient cannot be reached by phone after at least 3 contact attempts, a formal letter will be mailed out for the patient to call the HC and reschedule their appointment. The HC will continue attempts to reach the patient and reschedule the appointment. If after 2 weeks, the patient is still unable to be reached, the HC will discuss the situation with the pharmacist to decide what to do.

B. Patient Telehealth Encounters

1. Pharmacist visits will take place via videoconference using the VSee HIPAA Messenger online service. The HC will be with the patient at their home to assist with pre-visit preparation (outlined below in B.2) and provide technical support. The pharmacists will access VSee through their

assigned accounts on their workstation in the patient exam rooms (for MTMC) or by iPad (Mile Square, Family Medicine). All will be equipped with a web camera. The HC will facilitate the conference at the patient's home with an iPad.

2. HC Preparation: The HC will be responsible for helping the patient prepare for their pharmacist visit in the following ways:

    a. Review medications with patient: Prior to the pharmacist visit, the HC will use the patient's active medication list in the EMR, as well as clinical notes and fill history to review their medications with them. This may be done during any HC visit prior to the pharmacist visit. ***Note: A formal medication reconciliation will be done by the pharmacist, NOT the HC.***

    b. Gather medications: The HC will arrive 30 minutes prior to assist the patient in gathering any bottles or vials of medication to present to the pharmacist during their visit. This may include alternative therapies, supplements, and over-the-counter medications, etc. Also, both patient and HC will review which medications are taken and not taken (with barriers to adherence), to share with pharmacist.

    c. Prepare the patient for questions: The HC may help prepare the patient to respond to questions the pharmacist might have about medications, including name and dosage, etc. Additionally, the HC may help the patient prepare any questions *they* might have for the pharmacist.

    d. Self-monitored Blood Glucose (SMBG) readings: 2 weeks prior to the date of the pharmacist videoconference, the HC will send text message reminders to the patient to check their blood sugars and log them on paper. The HC will use the VSee chat function to send a photo of the *de-identified* log results. Despite requesting that the patient check their blood sugars, some patients may not adhere to this request. The lack of data may impact the effectiveness of the telehealth visit. Initial visits with the pharmacist will be conducted regardless of availability of the blood sugar readings. Follow-up visits that are specifically focused on medication changes may be cancelled at the pharmacists discretion when blood sugar readings are not available to guide medication changes.

e. Blood pressure: The HC will measure blood pressure (BP) prior to the start of the videoconference and will use the VSee chat function to send the reading to the pharmacist.

3. Videoconference initiation: The pharmacist will log in to VSee and prepare the webcam and software for a call. For additional information on opening VSee, beginning a call, and key contact information, please see the VSee/Telehealth Quick Guide (Appendix B). The HC assigned to the patient will be listed in the Comments section of the appointment along with any other pertinent details (e.g. language preference). The *pharmacist* will initiate the video call in VSee. The HC may call the MTM main line at XXX-XXX-XXXX to let the pharmacist know they are ready or if they are having connectivity or logistical issues.
4. Pharmacist Videoconference Visits may include the following evaluation:
   a. Review of medication orders and chart documentation, and perform medication reconciliation
   b. Review alternative and complementary medicine use
   c. Assess adherence to drug regimen and medication reconciliation
   d. Review of primary physician treatment plan or algorithm, including goals of therapy (algorithms include ADA, JNC 8, ACC/AHA, etc.)
   e. Discussion medication access issues, including financial barriers.
   f. Assessment of current conditions (diabetes, hypertension, hyperlipidemia) and self-monitoring data (including SMBGs and BPs)
   g. Pharmacist data assessment includes, but is not limited to, reviewing progress note, previous hospitalizations, and flowsheet results, and evaluating patient therapy.
5. Post visit reinforcement: The HC will reinforce any new recommendations and instructions provided by the pharmacist. This will include immediate post-visit discussion, as well as text or phone reinforcement after the visit within the following 3-5 days.
6. All patients will be seen based on the patients' medical needs and preferences, at least one pharmacist visit every 6 months (recommended alternating with HC visits).
7. The clinical pharmacist can perform the following functions as dictated by University protocols or

given by a prescriber verbally or in writing:

a. Adjust medication regimens in accordance to their drug response and therapy goal (e.g., high blood pressure, titrate medication dose)
b. Simplify therapy such as use of combination oral medication when appropriate
c. Authorize appropriate refills
d. Order appropriate laboratory tests (e.g., electrolytes, renal function)
e. Advise and plan in conjunction with their primary physician changes in therapy and monitoring parameters
f. Use an autonomy supportive approach
    i. Ask if willing to take before prescribing
    ii. Offer choices of therapy when appropriate

C. Patient Education Procedures:

1. Adequate education is paramount to successful pharmacist visits. The intent is for each patient to develop an understanding of their own care, including the following:
    a. Their disease states and the role of their medications
    b. Name, description, and purpose of each drug
    c. Time, strength, and method of administration
    d. Potential food and drug interactions
    e. Potential side effects they may experience
    f. Disease state goals. (e.g., BP goal < 130/80, A1c < 7%, LDL < 100)
    g. Use blood glucose, weight, peak flow, blood pressure logs, etc. to help the patient monitor their disease
    h. Lifestyle modifications that can be made (eating habits, physical activity, based on ADA guidelines)
    i. Use of a pill-box and other adherence aids (low literacy medication chart)
    j. Involve caregiver/family and HC in educational process
2. Patient’s understanding to the above will be assessed at each encounter - if patient’s knowledge base is low, he/she will be reeducated or provided reinforcement

3. Use an autonomy supportive or collaborative approach
    a. Identify sources of social support and community resources
    b. Acknowledge health beliefs and values
    c. Discuss appointment and other preferences in receiving health care services

D. Physician Contact:

1. The pharmacist's clinic note from the initial videoconference with the patient will be forwarded to their primary care physician (PCP) to notify them that the patient is a participant in the study.
2. Thereafter, a pharmacist will contact the primary provider whenever input is necessary.
3. As alternatives, the physician covering for the primary physician, the medical director of the clinic, or the PI (Dr. Gerber) is contacted.
4. As a last resort, the patient is directed to the UI Health Emergency Department
5. All patient interventions where physician input is necessary will be forwarded to the primary provider for review and medical oversight and/or the clinic medical director
6. The pharmacist will assist the primary provider with coordination of care involving medication use (e.g., specialist prescriptions, to avoid duplication of therapy or avoidable drug-drug interactions)

E. Patient Records:

1. *Pharmacist documentation* - At each visit, the ***pharmacist*** will be responsible for the following:
    a. A documented plan of care including goals of therapy, in line with those set by the primary physician, will be present in the electronic medical record (EMR).
    b. A progress note including all subjective and objective findings will be placed in the patient's EMR at each pharmacist videoconference visit.
    c. Progress notes are forwarded after the initial videoconference and as needed to the primary care physician.
    d. Medications and problem list will be reviewed and updated at each patient encounter.
    e. Possible drug or food allergies will be noted in the EMR.
    f. Documenting attendance at the encounter (e.g., any family members attending)
2. *HC documentation* - At each pharmacist visit, the ***HC*** will be responsible for the following:
    a. A documented visit summary, including any post-pharmacist videoconference visit

discussion with the patient that reinforced any concerns, orders, or instructions from the pharmacist. The HC will document this in a *HC Encounter* clinic note in the EMR.

b. The HC is NOT responsible for documentation on medication reconciliation, plan or goals of *medication therapy* as this will be captured by the pharmacist.

c. Encounter notes may be forwarded to the primary pharmacist and primary provider inbox when necessary.

F. Laboratory:

1. Since the visits will take place remotely from the patient's home, no point-of-care lab testing will be completed at the visit.
2. The patient will need to come to UI Health if the pharmacist or physician has ordered any tests. The HC may help reiterate this to the patient and help facilitate a visit to the UI Health Outpatient Laboratory if necessary.
3. Blood will be drawn for A1c and a full lipid panel tests at baseline and at 6, 12, 18, and 24 month data collection visits at the CRC. The blood will be processed at PCL-Alverno labs and results will be posted on their web-based results system. The HC will communicate results to the patient and pharmacist as needed. For this study, lab values will *not* be entered into the EMR.

G. Communication with HC:

1. HCs will route encounter notes to pharmacist as necessary to keep them informed of relevant information regarding barriers to goals and medication management.
2. Notes from the HC and the pharmacist may be routed to primary care physician if necessary.
3. Other methods (phone, encrypted email or EMR message center) may be used to discuss patient issues that are too complex for a HC encounter note. A phone call to the patient's primary pharmacist is the preferred method for discussing any issues that are highly sensitive.
4. In-person meetings between HCs and pharmacist team will occur monthly.

H. Cross-Coverage Procedures:

1. During periods when the primary clinical pharmacists are not available (i.e., vacation, conferences, teaching, etc.), the clinic will be covered by pharmacy faculty members who are familiar with the clinic procedures and have been added to the IRB protocol.
