# Supplementary material for: Mobile Health Intervention in Patients With Type 2 Diabetes: A Randomized Clinical Trial
Source: JAMA Netw Open. 2023 Sep 29;6(9):e2333629. doi: 10.1001/jamanetworkopen.2023.33629 (PMC10543137; doi:10.1001/jamanetworkopen.2023.33629)
Supplement: Supplement 2. — eFigure. Schematic Representation of Intervention Components eTable 1. Comparison of Characteristics Between Randomized vs Screened But Not Eligible/Not Randomized Individuals eTable 2. Comparison of Health Coach and Pharmacist Contact by Group eTable 3. Descriptive Statistics for Primary and Secondary Outcomes eTable 4. Summary of Clinician Encounters and Diabetes Medication Intensifications by Treatment Received eTable 5. Correlations Between Hemoglobin A1c Change and Diabetes Self-Management Behaviors, Diabetes Medication Intensification, and Clinician Encounters During Intervention Year [file jamanetwopen-e2333629-s002.pdf]

Supplementary Online Content

Gerber BS, Biggers A, Tilton JJ, et al. Mobile health intervention in patients with type 2 diabetes: a randomized clinical trial. *JAMA Netw Open*. 2023;6(9):e2333629. doi:10.1001/jamanetworkopen.2023.33629

eFigure. Schematic Representation of Intervention Components

eTable 1. Comparison of Characteristics Between Randomized vs Screened But Not Eligible/Not Randomized Individuals

eTable 2. Comparison of Health Coach and Pharmacist Contact by Group

eTable 3. Descriptive Statistics for Primary and Secondary Outcomes

eTable 4. Summary of Clinician Encounters and Diabetes Medication Intensifications by Treatment Received

eTable 5. Correlations Between Hemoglobin A_{1c} Change and Diabetes Self-Management Behaviors, Diabetes Medication Intensification, and Clinician Encounters During Intervention Year

This supplemental material has been provided by the authors to give readers additional information about their work.

© 2023 Gerber BS et al. *JAMA Network Open*.

eFigure. Schematic Representation of Intervention Components

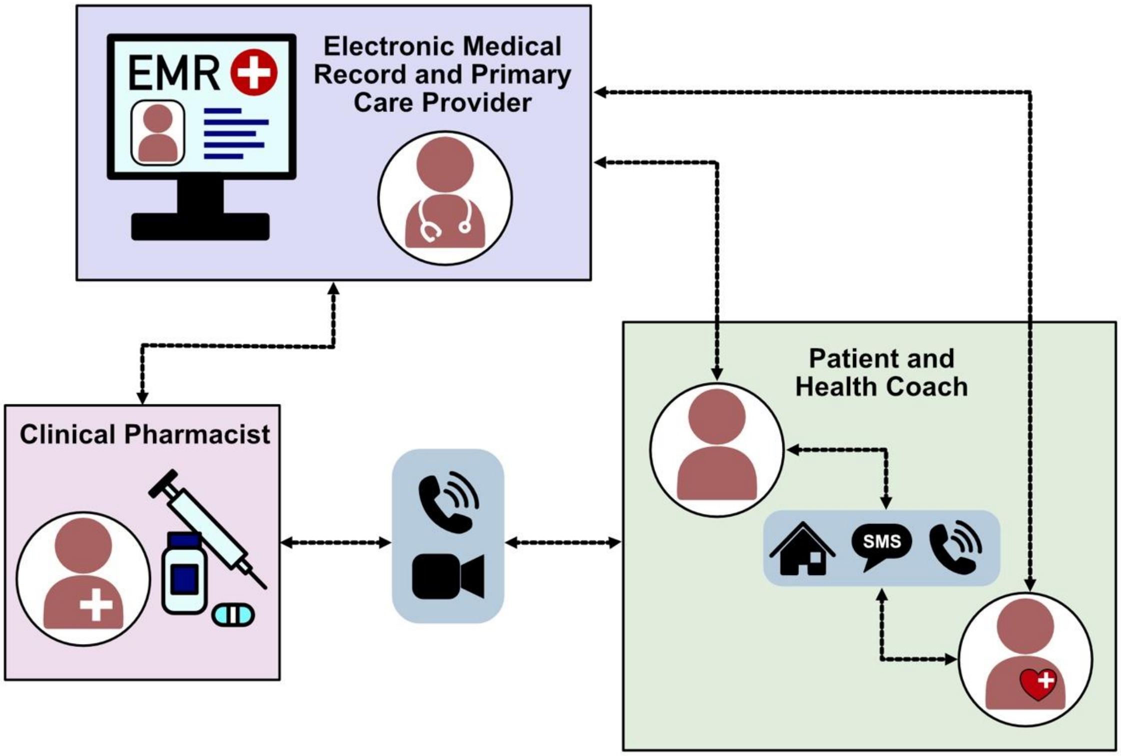

Pharmacist encounters occurred remotely via telehealth. Health coaches inside the patient's home (pre-COVID pandemic) facilitated the videoconference via internet-enabled iPad with cellular plan. During COVID, 3-way calling (audio or video) was conducted. Pharmacists reviewed patient medical records, including lab results, progress notes and medication lists, documented telehealth encounters, and communicated with PCPs through EMR secure messaging and progress note forwarding. Health coaches contacted patients at least monthly and conducted home visits (targeting every other month), text messaging (SMS), and phone calls, including facilitation of telehealth with pharmacists on alternating visits. Similar to pharmacists, coaches documented patient encounters in the EMR and communicated as needed with PCPs.

© 2023 Gerber BS et al. *JAMA Network Open*.

eTable 1. Comparison of Characteristics Between Randomized vs Screened But Not Eligible/Not Randomized Individuals

**Characteristic**	**Randomized** N = 221	**Not Eligible/Not Randomized** N = 288	**p-value**[a]
Age, Mean(SD) years	55.2 (9.5)	55.1 (11.1)	0.76
Unknown	0	10	
Race/Ethnicity, N(%)			0.03
Black or African American	148 (67%)	164 (58%)	
Latino or Hispanic	73 (33%)	121 (42%)	
Unknown	0	3	
Sex, N(%)			0.44
Male	67 (30%)	95 (34%)	
Female	154 (70%)	188 (66%)	
Unknown	0	5	

[a] Wilcoxon rank sum test (continuous); Pearson's Chi-squared test (categorical)

© 2023 Gerber BS et al. *JAMA Network Open*.

eTable 2. Comparison of Health Coach and Pharmacist Contact by Group

Contact Type	Intervention Group<br>N	Wait-list Control Group<br>N	Total<br>N	Intervention Group<br>Mean (SD)	Wait-list Control Group<br>Mean (SD)	Total<br>Mean (SD)	P-value [b]
**Any Contact**	736	688	1424	6.75 (3.76)	6.14 (4.03)	6.44 (3.90)	0.219
**Home Visit: Health Coach only**	316	217	533	2.90 (1.69)	1.94 (1.93)	2.41 (1.87)	<0.001
**Videoconference: Pharmacist/Health Coach**	285	234	519	2.61 (2.36)	2.09 (2.13)	2.35 (2.26)	0.072
**Phone Call: Health Coach**	89	210	299	0.82 (1.12)	1.88 (2.44)	1.35 (1.97)	0.002
**Clinic Visit with Health Coach [a]**	39	21	60	0.36 (1.00)	0.19 (0.59)	0.27 (0.82)	0.034
**Other/Unknown**	7	6	13	0.06 (0.28)	0.05 (0.23)	0.06 (0.25)	0.949

[a] Included more than 15 minutes spent with Health Coach
[b] Kruskal-Wallis test

© 2023 Gerber BS et al. *JAMA Network Open*.

eTable 3. Descriptive Statistics for Primary and Secondary Outcomes

Outcome	Time (mo)	Sample Size Total (Intervention/ Wait-List Control)	Intervention Group Mean (SD)	Wait-list Control Group Mean (SD)
**Hemoglobin A1c (%)**	0	221 (109/112)	9.22 (1.49)	9.24 (1.58)
	6	195 (93/102)	8.59 (1.54)	9.06 (1.80)
	12	183 (84/99)	8.41 (1.45)	9.05 (1.84)
	18	183 (86/97)	8.67 (1.79)	8.56 (1.85)
	24	170 (80/90)	8.51 (1.66)	8.47 (1.85)
**Systolic blood pressure (mm Hg)**	0	221 (109/112)	127.39 (16.01)	129.29 (16.66)
	6	185 (91/94)	129.39 (15.66)	130.99 (17.92)
	12	146 (70/76)	127.76 (17.09)	132.01 (18.02)
	18	132 (68/64)	133.09 (17.37)	131.70 (19.92)
	24	114 (55/59)	130.36 (19.97)	130.35 (16.04)
**Diastolic blood pressure (mm Hg)**	0	221 (109/112)	76.86 (8.51)	77.32 (9.96)
	6	185 (91/94)	77.53 (9.19)	78.49 (9.03)
	12	146 (70/76)	76.17 (9.65)	78.94 (9.26)
	18	132 (68/64)	79.49 (7.44)	78.53 (8.70)
	24	114 (55/59)	77.39 (10.09)	77.93 (8.15)
**Total cholesterol (mg/dL)**	0	221 (109/112)	160.45 (43.84)	169.89 (49.54)
	6	190 (93/97)	162.97 (46.33)	170.24 (47.37)
	12	171 (79/92)	159.15 (38.44)	165.20 (45.53)
	18	165 (82/83)	164.28 (49.60)	158.27 (46.15)
	24	151 (70/81)	160.07 (45.54)	170.64 (46.93)
**HDL cholesterol (mg/dL)**	0	221 (109/112)	46.14 (12.85)	44.47 (10.70)
	6	190 (93/97)	44.98 (11.22)	44.60 (11.27)
	12	171 (79/92)	45.76 (11.82)	44.39 (11.40)
	18	165 (82/83)	46.94 (13.66)	43.84 (10.30)
	24	151 (70/81)	46.67 (13.01)	43.79 (11.58)
**LDL cholesterol (mg/dL)**	0	221 (109/112)	85.72 (37.96)	93.52 (40.23)
	6	190 (93/97)	87.28 (39.85)	92.91 (38.77)
	12	170 (79/91)	82.76 (34.19)	87.63 (36.03)
	18	165 (82/83)	85.70 (37.85)	81.84 (36.19)

© 2023 Gerber BS et al. *JAMA Network Open*.

	24	150 (70/80)	84.09 (39.84)	91.44 (38.67)
**Triglycerides (mg/dL)**	0	221 (109/112)	143.65 (72.32)	167.57 (127.60)
	6	190 (93/97)	157.71 (140.39)	178.75 (128.80)
	12	171 (79/92)	160.71 (103.41)	176.71 (147.57)
	18	165 (82/83)	159.21 (128.97)	169.69 (117.87)
	24	151 (70/81)	149.34 (70.94)	184.88 (138.84)
**Body Mass Index (kg/m^2)**	0	213 (106/107)	34.92 (8.64)	36.35 (9.29)
	6	174 (89/85)	35.18 (8.48)	36.53 (9.28)
	12	141 (69/72)	34.35 (8.29)	37.03 (9.46)
	18	125 (64/61)	34.14 (8.55)	36.51 (8.58)
	24	108 (53/55)	32.83 (8.77)	36.89 (8.80)
**Diabetes distress (DDS)**	0	220 (109/111)	2.90 (1.54)	3.13 (1.45)
	6	201 (96/105)	2.46 (1.35)	2.85 (1.33)
	12	195 (92/103)	2.55 (1.42)	2.76 (1.35)
	18	189 (90/99)	2.58 (1.40)	2.73 (1.36)
	24	180 (86/94)	2.38 (1.36)	2.66 (1.33)
**Diabetes self-efficacy**	0	220 (109/111)	6.89 (1.89)	6.86 (1.87)
	6	202 (96/106)	7.33 (1.76)	7.02 (1.71)
	12	193 (91/102)	7.23 (2.05)	6.95 (1.83)
	18	189 (90/99)	7.34 (1.93)	7.57 (1.73)
	24	180 (86/94)	7.31 (2.13)	7.36 (1.85)
**Depression (PHQ-9) score**	0	220 (109/111)	5.85 (5.39)	5.97 (4.99)
	6	199 (94/105)	4.97 (4.86)	6.15 (5.87)
	12	193 (91/102)	4.78 (5.15)	5.40 (5.31)
	18	187 (90/97)	4.99 (5.05)	5.06 (4.98)
	24	174 (83/91)	4.22 (5.29)	5.19 (5.43)
**Diabetes social support**	0	221 (109/112)	15.17 (4.58)	14.65 (4.67)
	6	202 (97/105)	16.33 (3.52)	14.94 (4.42)
	12	194 (91/103)	16.26 (4.30)	15.84 (4.42)
	18	188 (90/98)	16.56 (3.50)	16.60 (3.94)
	24	180 (86/94)	16.62 (3.86)	16.83 (3.52)
**Medication taking**	0	221 (109/112)	82.75 (25.34)	80.62 (26.21)
	6	203 (97/106)	89.18 (17.36)	84.43 (23.01)

© 2023 Gerber BS et al. *JAMA Network Open*.

	12	195 (92/103)	91.20 (14.96)	85.92 (20.74)
	18	189 (90/99)	90.56 (15.39)	85.66 (22.14)
	24	180 (86/94)	89.77 (18.34)	88.30 (19.49)
**Diabetes self-care, diet score**	0	221 (109/112)	3.32 (2.26)	3.51 (2.05)
	6	203 (97/106)	4.13 (2.10)	4.09 (1.98)
	12	195 (92/103)	4.39 (2.04)	4.19 (2.17)
	18	189 (90/99)	4.32 (1.94)	4.68 (2.01)
	24	180 (86/94)	4.36 (1.99)	4.48 (1.95)
**Diabetes self-care, exercise score**	0	221 (109/112)	2.52 (2.34)	2.41 (2.14)
	6	203 (97/106)	2.88 (2.20)	2.46 (2.25)
	12	195 (92/103)	2.77 (2.32)	2.51 (2.16)
	18	189 (90/99)	2.67 (2.18)	2.86 (2.32)
	24	180 (86/94)	3.13 (2.47)	2.38 (2.09)
**Diabetes self-care, glucose testing score**	0	219 (108/111)	3.81 (2.71)	3.45 (2.82)
	6	202 (97/105)	4.75 (2.53)	3.82 (2.70)
	12	194 (92/102)	4.58 (2.75)	4.28 (2.80)
	18	188 (90/98)	4.54 (2.75)	4.53 (2.63)
	24	180 (86/94)	4.60 (2.83)	4.73 (2.60)
**Quality of life**	0	221 (109/112)	68.44 (22.23)	67.30 (20.32)
	6	203 (97/106)	72.05 (23.20)	70.00 (20.80)
	12	195 (92/103)	72.27 (20.83)	68.64 (21.67)
	18	189 (90/99)	70.87 (22.22)	70.53 (20.05)
	24	180 (86/94)	74.90 (21.12)	71.59 (23.93)

© 2023 Gerber BS et al. *JAMA Network Open*.

eTable 4. Summary of Clinician Encounters and Diabetes Medication Intensifications by Treatment Received

	Clinician Encounters[a]					Intensifications[b]				
	Usual Care		Intervention			Usual Care		Intervention		
Months	Mean	SD	Mean	SD	P-value[c]	Mean	SD	Mean	SD	P-value[c]
0-6	1.17	1.41	1.30	1.51	0.57	0.73	0.94	1.00	1.40	0.24
6-12	1.40	1.60	1.21	1.47	0.30	0.63	0.94	0.61	1.08	0.37
12-18	1.28	1.63	1.60	1.59	0.06	0.61	1.03	0.88	1.22	0.06
18-24	1.45	1.44	1.62	1.54	0.43	0.45	0.84	0.53	0.92	0.55

[a] Clinician encounters billed as CPT4 99201-99205 and 99211-99215; HCPCS G0463, G0438, G0439 (does not include intervention pharmacist)

[b] Intensification defined as an increase in dose or number of therapeutic classes (prescriptions written within 12 months before enrollment served as baseline)

[c] Mann-Whitney U test

© 2023 Gerber BS et al. *JAMA Network Open*.

eTable 5. Correlations Between Hemoglobin A_{1c} Change and Diabetes Self-Management Behaviors, Diabetes Medication Intensification, and Clinician Encounters During Intervention Year[a]

Behavior	Intervention Group Treatment			Wait-list Control Group Treatment			Overall Treatment		
	n	r	P-value	n	r	P-value	n	r	P-value
Medication Adherence[b]	84	-0.23	0.04	86	-0.14	0.20	170	-0.18	0.02
General Diet[b]	84	-0.01	0.94	86	-0.18	0.10	170	-0.11	0.16
Exercise[b]	84	-0.34	<.01	86	-0.05	0.62	170	-0.20	<.01
Blood Glucose Testing[b]	83	-0.04	0.71	86	-0.29	<.01	169	-0.18	0.02
Diabetes Medication Intensification[c]	84	-0.04	0.73	86	-0.16	0.15	170	-0.11	0.17
Clinician Encounters[d]	84	0.04	0.75	86	-0.09	0.44	170	-0.03	0.72

[a] Spearman's correlations with two-sided P-values

[b] Subscale of the Summary of Diabetes Self-Care Activities (SDSCA)

[c] Intensification defined as an increase in dose or number of therapeutic classes (prescriptions written within 12 months before enrollment served as baseline)

[d] Clinician encounters billed as CPT4 99201-99205 and 99211-99215; HCPCS G0463, G0438, G0439 (does not include intervention pharmacist)

© 2023 Gerber BS et al. *JAMA Network Open*.
