# Supplementary material for: Mobile Health Intervention in Patients With Type 2 Diabetes: A Randomized Clinical Trial
Source: JAMA Netw Open. 2023 Sep 29;6(9):e2333629. doi: 10.1001/jamanetworkopen.2023.33629 (PMC10543137; doi:10.1001/jamanetworkopen.2023.33629)
Supplement: Supplement 3. — Data Sharing Statement [file jamanetwopen-e2333629-s003.pdf]

Data Sharing Statement

Gerber. Mobile Health Intervention in Patients With Type 2 Diabetes. *JAMA Netw Open*. Published September 29, 2023. doi:10.1001/jamanetworkopen.2023.33629

Data
Data available: Yes
Data types: Deidentified participant data, detailed data dictionary/codebook
How to access data: Data and associated metadata will be shared through UIC's institutional repository, INDIGO, hosted by Figshare. The dataset DOI is 10.25417/uic.24002007 and can be accessed directly via: https://indigo.uic.edu/
When available: With publication

Supporting Documents
Document types: ReadMe file, R Statistical/analytic code, a blank Informed consent form, and the study protocol document.
How to access documents: Supporting documentation will be shared alongside the data prior to publication in UIC's institutional repository, INDIGO, findable by the same DOI: 10.25417/uic.24002007
When available: With publication

Additional Information
Who can access the data: Anyone requesting the data
Types of analyses: Any purpose
Mechanisms of data availability: Without investigator support
